# “I was trying to save the world”: delusion-like ideation and associated impacts reported by Western practitioners of Buddhist meditation

**DOI:** 10.3389/fpsyg.2025.1644684

**Published:** 2025-10-24

**Authors:** Elizaveta Solomonova, Jared R. Lindahl, Ian Gold, David J. Cooper, Charlotte Little, Damian Arteca, Chenxi Cao, Willoughby B. Britton

**Affiliations:** ^1^Neurophilosophy Lab, McGill University, Montreal, QC, Canada; ^2^Department of Philosophy, McGill University, Montreal, QC, Canada; ^3^Department of Religious Studies, Brown University, Providence, RI, United States; ^4^Department of Psychiatry and Human Behavior, Warren Alpert Medical School, Brown University, Providence, RI, United States

**Keywords:** meditation-related challenges, delusional ideation, mental health, meditation and culture, meditation-induced psychosis

## Abstract

Delusional ideation is characteristic of psychopathology (e.g., psychosis, bipolar disorder) and is also found among the general population. Contemporary case studies have documented delusional ideation as a feature of meditation-induced psychosis, and Buddhist literature on the side effects and adverse effects of meditation also includes discussion of transient experiences that could be considered delusional or delusion-like ideation. Drawing upon interviews with more than 100 Buddhist meditation practitioners and meditation experts (teachers and clinicians) in the West, this paper presents a mixed-methods study of delusion-like ideation (DLI) associated with meditation. We establish a typology of eight types of DLI and report their relative frequencies among the sample; we identify impacts and treatment outcomes associated with DLI; and we provide four case studies that illustrate the risk factors, trajectories, outcomes, and appraisals associated with DLI. We show how responses to DLI are shaped not only by the type of DLI but also by their duration, severity, and impact, as well as the associated appraisals made both by meditators and by meditation teachers and psychiatrists. In some cases, the phenomenology of DLI suggests influences from the lived context of Buddhist meditation cultures. Furthermore, although DLI are normalized in Buddhist meditation culture under certain circumstances, meditation experts also noted the potential severity of meditation-related DLI, with some identifying it as a “red flag” meriting close monitoring if not immediate intervention. Finally, we discuss various explanatory models that could account for the presence, content, and impacts of DLI among meditators, drawing upon the environmental conditions and social contexts of meditation retreats, the role of attention and sensory attenuation in meditation practice, and the ways in which meditation-related DLI can function as a cultural and spiritual “idiom of distress.”

## Introduction

Meditation practices have become commonplace in the West. Meditation techniques can be encountered both in religious and spiritual centers as well as in wellness-oriented and healthcare settings. The vast majority of research in the emerging field of contemplative science has documented a variety of benefits of meditation associated with both religious practices as well as contemplative practices adapted for and applied in clinical contexts. More recently, however, a growing literature has begun to historicize and problematize the relationship between meditation and mental health outcomes. Numerous studies have now documented multiple types of challenges and adverse effects associated with meditation and mindfulness practices (Britton et al., 2021; Farias et al., 2020; Goldberg et al., 2021; Lambert et al., 2021; Lindahl et al., 2017; Pauly et al., 2022; Sparby et al., 2024; Taylor et al., 2022; Van Dam et al., 2025). This complicates the often oversimplified claims about the benefits of meditation being universal and without risk. Concurrently, historians and scholars of religious studies have argued that meditation needs to be understood as a contextual and social practice. This research has called attention to the historical conditions that led to the application of meditation towards Western notions of well-being, while also contrasting the modern theory and practice of meditation with the more diverse range of effects and goals found throughout the history of religions, especially Buddhism (Hickey, 2019; McMahan, 2008; McMahan and Braun, 2017; Salguero, 2023; Wilson, 2014).

Building upon previous research on meditation-related challenges and adverse effects as well as case reports on meditation-related psychosis, this paper provides an analysis of reports of delusion-like ideation (DLI) and its impacts as described by meditation practitioners and meditation experts from the *Varieties of Contemplative Experience* project (Lindahl et al., 2017). We establish a typology of meditation-related DLI and draw upon qualitative data from practitioner narratives to illuminate the trajectory of various DLI experiences, including how practitioners and experts describe, assess and evaluate them, as well as the short and long-term effects DLI can have on meditation practice and mental health. We present quantitative data on the relative prevalence of different types of DLI reported in this sample of meditators as well as on the impacts and treatment responses meditators sought. We discuss these findings in light of growing epidemiological data and current theories of delusional ideation. This literature stresses that delusions are common in non-psychotic populations, are varied in terms of their impact (from benign unusual ideas to prodromal symptoms of a psychotic disorder), and that delusional ideation is rooted in relational contexts of social and cultural life. We conclude by presenting suggestions for further research on the complexity of psychological responses to intensive Buddhist meditation practices and consider the etiology and development of DLI in the context of challenging spiritual or existential experiences more broadly.

### Delusions and delusional ideation

Delusions are defined in the Diagnostic and Statistical Manual of Mental Disorders (DSM-5-TR) as “fixed beliefs that are not amenable to change in light of conflicting evidence.” Those bizarre beliefs must also be “implausible and not understandable to same-culture peers” (e.g., it is not an article of religious faith) (American Psychiatric Association, 2022). This rather broad definition potentially encompasses a vast variety of phenomena, many of which would not necessarily be considered delusional (e.g., religious beliefs, conspiracy theories). Multiple classification systems of delusions exist, but the main categories, forms, or types of delusions appear to be present across cultures and time periods (Gold and Gold, 2015; Grunfeld et al., 2023). Cross-culturally, the most common types of delusional ideation are delusions of persecution (e.g., that other people, governments, groups or entities are planning to harm the individual), delusions of reference (e.g., thinking that events in the world, objects or other people’s gestures are signs that are communicative and are directed at the individual), and grandiose delusions (e.g., thinking that the individual has special powers or talents, or is distinguished compared to other people) (American Psychiatric Association, 2022).

While delusions are a hallmark of schizophrenia spectrum disorders as well as acute psychotic episodes, including transient ones, a growing body of research suggests that delusional ideation is highly prevalent in non-clinical populations, with estimates varying between 10 and 30% (Freeman, 2006). Recent theories suggest that different kinds of delusional ideation are located on a continuum between mild and severe (Betts et al., 2018) and represent multifactorial (Yung and Lin, 2016) and dynamic rather than all-or-nothing or static phenomena. It has also been suggested that delusional ideation represents a measure of psychosis-proneness, i.e., a potential predisposing trait to psychosis, in particular when a high degree of delusional thought is accompanied by hallucinatory phenomena and other psychotic-like experiences (Kelleher et al., 2011; Smeets et al., 2013). At the same time, transient delusional ideation in the general population, especially when referring to experiences typically understood as paranormal or irrational, has also been suggested to express “healthy schizotypy” (McCreery and Claridge, 2002). Some delusional experiences may even represent adaptive ways of coping with existential challenges by providing an alternative meaning-making container to help regain a sense of agency over one’s own life (Bortolotti, 2023).

The dominant theoretical accounts typically appeal to one or both of two factors in the etiology of delusion: “anomalous” experience (Maher, 1974, 1999) (Ellis and Young, 1996) and some form of disorder of thinking or reasoning. However, recent scholarship has advocated for a theoretical reorientation concerning delusions, highlighting how they are rooted in alterations in social functioning and social cognition (Bell et al., 2021; Bortolotti, 2023; Gold and Gold, 2015). Bell and colleagues argue that some delusions and other kinds of irrational beliefs may even be socially adaptive, socially transferred and understood as cognitions associated with social coalitions. Indeed, the most prevalent delusions have social themes and reflect imaginary relations with others. Delusions of persecution require imagined participation of other people; delusions of reference suggest that other people are leaving secret messages for the individual; and delusions of grandiosity presuppose that there is a comparison between the individual’s special ability and their peer group. In non-psychotic individuals, delusional ideation is strongly associated with intensified social imagery, including felt presence, loneliness, social anxiety and facets of empathy (Robertson et al., 2024). Further, emerging work from interdisciplinary approaches to the phenomenology of delusional and hallucinatory experiences shows that while the *types* of delusions (persecution, grandiosity, etc.) may be relatively invariant, specific delusional or hallucinatory *content* is responsive to and influenced by environmental factors, such as lived experience, social norms, culture and expectations (Gold and Gold, 2015; Grunfeld et al., 2023; Laroi et al., 2014; Suhail and Cochrane, 2002). This suggests that delusional ideation should be understood in relation to rooted in personal context and in lived experience.

From the perspective of Western psychiatry, religious or spiritual practices have been identified as both risk and protective factors in the development of delusions. Religious or spiritual context can provide normative explanatory frameworks for strange experiences through paranormal, supernatural, or spiritual appraisals. For example, in a systematic literature review, Gearing and colleagues report that 27 studies (37% of studies examined by the review) identified religion as a risk factor for delusions and hallucinations, potentially contributing to adverse outcomes and negative experiences (such as being cursed, being controlled by Satan, experiencing strong feelings of having sinned, etc.). At the same time, the authors also report that 20 studies (34% of studies in their review) identified religion as a protective factor in the development of delusions, with religion providing context, meaning and coping mechanisms, and facilitating better outcomes for patients with schizophrenia (Gearing et al., 2011). Despite acknowledging the impact of culture on mental health disorders and the need for culturally sensitive interventions, DSM-5 retains the adherence of previous editions to the Western psychological tradition as a normative framework for understanding mental health and the human mind in general (Murphy, 2015). Still, the ideas of cultural formulation and cultural concepts of distress that are included in the DSM-5 allow for local forms of understanding to be applied to various kinds of symptomatology, including delusions.

### Delusional ideation in Buddhist literature on meditation practices

Buddhist texts describe a range of effects, both positive as well as challenging and adverse, that can arise through the practice of meditation (Lindahl et al., 2019). For instance, in his treatise *The Vajra Essence*, Düdjom Lingpa, a nineteenth-century Tibetan Buddhist, catalogues a range of transient “meditation experiences” (Tib. *nyams*) that can arise from concentration practice (Lingpa, 2015). Some of these bear a resemblance to aspects of delusional ideation. For example, one *nyams* is “an inexplicable sense of paranoia about meeting other people, visiting their homes, or being in town” (Lingpa, 2015, p. 23). This paranoid feeling can be further amplified and turned into an “unbearable anger due to having paranoid thoughts that everyone is gossiping about you and disparaging you” (*Ibid.*, p. 24). Another *nyams* is related to delusions of reference and is a conviction that visual and auditory environmental stimuli have special meaning, are directed at the individual, and make them think that “that must be a sign or omen for me, and compulsively speculate about the chirping of birds and everything else you see and feel” (*Ibid.*, p. 23).

Similarly, the *Śūraṅgama Sūtra* (an eighth century apocryphal Mahāyāna *sūtra* that is particularly significant in East Asian forms of Buddhism) lists 50 “demonic states” (Chn. *mojing*) that can arise when the mind is concentrated. Some of these are distortions in perception and cognition, while many others resemble grandiosity. Recently, scholars have considered this text in the broader context of discussions of “meditation sickness” (Chn. *chanbing*) in East Asian Buddhism (Salguero, 2023). Consider the following excerpts:

In this state of *dhyāna* [meditative absorption], as form vanishes and receptiveness manifests, the practiser may think that he has achieved full realization. This illusion causes him suddenly, without any reason, to give rise to self-conceit so that he regards himself, though inferior, as equal to others; though equal, as superior to others and to superiors; as being a saint when he is not; and as not inferior to inferiors; all these feelings occur together. Even all the Buddhas are nothing to him (The Śūraṅgama Sūtra, 1966).

In this state of *dhyāna*, as form vanishes and receptiveness manifests, before new headway is made and after his previous experience has passed, he may find himself in a situation which seems very dreadful and full of danger, and causes him endless anxiety and perplexity. He seems to sit on a hot iron bed or to drink poisonous medicine. As a result, he tires of life and seeks to end it to get rid of this torment. This is practice without the (necessary) expedient method and, is harmless if he knows the cause. It is not a saintly state, but if he regards it as such, he will succumb to the demon of anxiety who will control his mind causing him to cut his own flesh with a sharp knife so that he can die or to flee to the mountains and groves in order to avoid other people (*Ibid.*, p. 286).

In the first excerpt the practitioner holds an erroneous belief in their accomplishments and a false sense of superiority, while in the second one he is tormented, full of anxiety and dread, and may act upon these thoughts and feelings. Both of these states are seen as undesirable consequences that follow from having a poor understanding of how to relate to unusual experiences: “Deluded and wayward practisers who do not know their capabilities, cannot distinguish these states when they manifest and wrongly declare that they have attained the holy rank” (p. 330).

### Delusional ideation and psychotic spectrum experiences and their relationship to meditation

The relationship between meditation and delusional ideation has already been investigated in two separate bodies of research. On the one hand, numerous case studies dating back decades have documented delusional ideation in the context of acute psychotic episodes linked to intensive meditation practice (Chan-Ob and Boonyanaruthee, 1999; Charan et al., 2023; Joshi et al., 2021; Kuijpers et al., 2007; Prakash et al., 2018; Sethi and Bhargava, 2003; Sharma et al., 2016). However, the precise role that specific meditation techniques versus other social and contextual factors play in the development of psychosis or of psychotic-like symptoms remains unclear (Sharma et al., 2019).

On the other hand, acceptance-based and mindfulness-based programs have been deployed in the treatment of psychosis. Systematic reviews and meta-analyses of mindfulness-based programs for psychosis have found a range of improvements for psychotic symptoms, with larger improvements for functioning and illness awareness and smaller improvements for the positive symptoms, such as delusions and hallucinations (Yip et al., 2024; Hodann-Caudevilla et al., 2020). However, a closer look at the literature casts doubt on the claim that meditation can be used to effectively treat psychosis. First, meditation practice in mindfulness-based programs for psychosis is limited to short durations (10 min) and are not required for homework, which has led researchers to question the importance of meditation in outcomes compared to other prominent factors including self-compassion, acceptance, psychoeducation, and disease management (Cabrera et al., 2024; Ellett, 2024). Second, while the safety profile of mindfulness-based programs appears favorable, less than half of trials have actively measured adverse effects, including side effects of treatment, deterioration of symptoms, or hospitalizations (O’Brien-Venus et al., 2024).

### Challenges associated with meditation and the *Varieties of Contemplative Experience* project

In recent years, scholars have increasingly called for a more nuanced and fine-grained understanding of meditation, as growing number of studies reveal diverse mechanisms across practices and a range of responses among different populations of practitioners. Growing evidence indicates that meditation practices can be associated with challenging experiences and adverse outcomes, and that, far from being a universal good, meditation may be harmful to some individuals under certain circumstances. Consequently, a nuanced and complex approach that takes into consideration methodologies, target groups and harm reduction is gradually emerging (Britton et al., 2021; Farias et al., 2020; Goldberg et al., 2021; Lambert et al., 2021; Van Dam et al., 2025; Van Dam et al., 2018).

The *Varieties of Contemplative Experience* (VCE) project (Lindahl et al., 2017) has been at the forefront of documenting meditation-related challenges, how they are understood in different contexts, and how meditation practitioners as well as meditation teachers and clinicians respond to them when they occur. The principal objective of the VCE study was to identify the range of challenging experiences associated with Buddhist meditation in the West, their risk factors and remedies, and how they were interpreted and understood (Lindahl et al., 2017). Subsequent qualitative analysis identified the criteria used for differentiating between normative appraisals (the challenging experience is an expected part of a spiritual or religious path) and psychopathological appraisals (the challenging experience is an indication of potential illness) or determining whether there is a need for intervention (Lindahl et al., 2020). Focused secondary analyses have illuminated the influence of worldviews (Lindahl et al., 2022b) and teachers (Canby et al., 2025) on the trajectory of meditation-related challenges, and have demonstrated how specific challenges such as the perception of lights (Lindahl et al., 2014), somatic sensations of “energy” (Cooper et al., 2021), re-experiencing of traumatic memories (Lindahl, 2017), changes in sense of self (Lindahl and Britton, 2019), and fear (Lindahl et al., 2022a) are understood in both biomedical and religious contexts.

### Delusion-like ideation defined for the present analysis

The type of meditation-related challenge most difficult for researchers to delimit and operationalize in the original VCE analysis was “delusional, irrational, or paranormal beliefs.” As noted in the initial findings of the VCE study, this type of experience, perhaps more than any other, was closely bound up with culturally specific worldviews and appraisals. In addition to thoughts or beliefs described by the interviewed meditation practitioners themselves as delusional or irrational in retrospect (e.g., disconfirmed by their appraisal of the evidence), this category also included thoughts or beliefs that may have seemed highly unusual or concerning to an authority in their culture or subculture, such as a meditation teacher, family member, or the meditation practitioners themselves. Finally, it also included thoughts or beliefs that would have been viewed as unusual or concerning in certain modern Western contexts, but that would be viewed as potentially normal or normative in traditions Buddhist contexts (e.g., communication with a disembodied teacher, or an insight into a past life) (Lindahl et al., 2017).

For the present analysis, we have operationalized this continuum of related experiences into multiple types of “delusion-like ideation” (DLI) in order to better capture the complexity of how they were interpreted and appraised across multiple contexts, both religious and biomedical. Our approach is to investigate the types of DLI, as well as their appraisals, trajectories of development, and impacts on mental health and meditation practice as reported by the meditation practitioners and as observed by the meditation experts interviewed in the VCE study. Thus, the use of DLI as a shorthand for these challenging experiences reflects our commitment to inclusivity of multiple epistemic systems and contexts of practice within which they arise.

### Objectives

The present analysis has the aim of describing the different types of DLI that Buddhist meditators and meditation experts in the West identified as challenges associated with meditation practice, as well as their impacts. We employ qualitative methods to establish a typology of DLI associated with meditation and to provide narrative examples of how they were understood by our participants and how they developed and changed over time. We also summarize and quantify the impacts and treatment responses for those reporting DLI. We employ quantitative methods to investigate demographic differences between those reporting DLI and those not reporting DLI.

## Materials and methods

The *Varieties of Contemplative Experience* (VCE) project is a mixed-methods study of meditation-related challenges (Lindahl et al., 2017). The study aims to identify (1) the types of experiences meditators report as either challenging, difficult, distressing, or impairing of functioning; (2) the factors that cause or influence the trajectory of meditation-related challenges; (3) how different challenges are interpreted within and beyond Buddhist communities; (4) how meditation practitioners navigate and respond to challenges when they arise; and (5) how meditation teachers guide their students when meditation-related challenges occur.

Purposive sampling led to the recruitment of meditation practitioners (n = 60) and meditation experts (n = 32). All meditation practitioners were in some way affiliated with a recognized Buddhist lineage in the West, and all practitioners had challenging, distressing, or functionally impairing experiences that they attributed solely to meditation or that were exacerbated by meditation. Meditation experts were either meditation teachers who had personally guided students through challenges or were clinicians who use meditation techniques therapeutically and were familiar with meditation-related challenges in both clinical and non-clinical applications. Some had dual training as both clinicians and meditation teachers. The initial study recruited practitioners equally (n = 20) from Theravāda, Zen, and Tibetan lineages, while a follow-up study added another 8 practitioners and 1 expert from the tradition of *vipassanā* meditation as taught by S.N. Goenka.

Semi-structured interviews with meditation practitioners queried the range of meditation-related challenges; what practitioners believed caused them or influenced their trajectory; how practitioners and other authorities (like meditation teachers and clinicians) interpreted them; and what remedies or responses were helpful or unhelpful for navigating them. Experts recounted the kinds of meditation-related challenges they had witnessed in their students or clients, how they interpreted them, and what remedies or responses they suggested for managing or mitigating them. The research methodology for this project was approved by the Brown University Institutional Review Board. For a comprehensive description of the original study methodology, participant demographics, and the phenomenology and influencing factors results, see Lindahl et al. (2017).

### Analyses of delusion-like ideation

#### Qualitative analyses

A combination of theory-driven (Gold and Gold, 2015) and data-driven coding was employed to establish a typology of delusion-like ideation. The eight distinct types of DLI were: (1) being controlled; (2) death or dying; (3) grandiosity; (4) ideas of reference; (5) misidentification; (6) paranoia: general; (7) paranoia: danger from others; and (8) special knowledge. A few miscellaneous references to DLI not meeting criteria for any of the above were coded as “other.” All references to DLI in the VCE dataset were reviewed by two authors (JL and DC), and any discrepancies were discussed until consensus was reached.

In addition, we coded for 13 kinds of impacts and treatment responses that followed meditation-related DLIs including whether participants: (1) received a formal diagnosis of a mental health disorder; (2) were admitted to a hospital; (3) were removed from or left the retreat; (4) were prescribed an anti-psychotic medication; 5() were prescribed another psychotropic medication; (6) continued meditation after the incident; (7) stopped meditating after the incident; (8) used alternative medicine as an intervention (e.g., homeopathy, acupuncture, herbal, massage therapy, etc.); (9) used grounding activities; (10) used yoga; (11) changed their diet; (12) saw a therapist; and (13) saw a psychiatrist outside of a hospital context. All outcomes were coded as binary yes/no variables. The classification of an item into impacts and responses was based on participants’ explicit mention in interview that those outcomes were directly related to their challenging DLI experience. The interview excerpts were coded by two coders (ES and CL), and consensus was established through a discussion.

#### Quantitative analyses

In the original mixed-methods VCE study, all meditation practitioners were invited to participate in a follow-up survey which asked them to provide more detailed information about: their demographic background; their practice background; the onset, duration, and severity of their meditation-related challenges; the remedies they found helpful and unhelpful; and the causal relationship between their challenges and their meditation practice (Lindahl et al., 2017). Many of these queries were fixed-choice options that facilitated additional quantitative analyses of differences between those reporting DLI and those who did not. Types of DLI were coded as binary yes/no variables, indicating a presence of one or more types of delusional ideation in participants’ narratives. Aside from their contribution to the count of total types of DLI, the few DLI coded as “other” were omitted from analyses due to their inherent heterogeneity (Tables 1, 2).

**Table 1:** Participant demographics: meditation practitioners.

	VCE Practitioners	DLI Sub-group	No-DLI Sub-group
Total Number	68	33	35
Gender	Male: 37	Male: 18	Male: 19
	Female: 31	Female: 15	Female: 16
Race	White: 60	White: 28	White: 31
	More than one race: 4	More than one race: 2	More than one race: 2
	Native American: 1	Native American: 1	Native American: 0
	Other: 3	Other: 2	Other: 1
Ethnicity	Non-Hispanic: 63	Non-Hispanic: 30	Non-Hispanic: 33
	Hispanic: 5	Hispanic: 3	Hispanic: 2
Practice Tradition	Theravāda: 28^a^	Theravāda: 17^a^	Theravāda: 11^a^
	Zen: 20	Zen: 7	Zen: 13
	Tibetan: 20	Tibetan: 9	Tibetan: 11
Is a Meditation Teacher	Yes: 29; No: 39	Yes: 10; No: 23	Yes: 19; No: 14
Age at Onset^b^	Under age 30: 28	Under age 30: 13	Under age 30: 15
	Over age 30: 38	Over age 30: 19	Over age 30: 19
	Mean age: 34.4	Mean age: 34.4	Mean age: 34.3
Regular Meditation Practice Prior to Onset^c^	Less than 10 years: 46	Less than 10 years: 19	Less than 10 years: 27
	More than 10 years: 21	More than 10 years: 14	More than 10 years: 7
	Mean: 6.8 years	Mean: 8.2 years	Mean: 5.3 years
Practice Intensity at Onset	Retreat only: 45	Retreat only: 24	Retreat only: 21
	Daily practice only: 11	Daily practice only: 4	Daily practice only: 7
	Both: 12	Both: 5	Both: 7
Practicing within a Community at Onset	Yes: 46; No: 22	Yes: 22; No: 10	Yes: 24; No: 11
Working with Teacher at Onset	Yes: 47; No: 21	Yes: 23; No: 11	Yes: 24; No: 11
Prior Psychiatric History: Any	Yes: 20; No: 48	Yes: 10; No: 23	Yes: 10; No: 25
Prior Psychiatric History: Bipolar or Schizophrenia	Yes: 3; No: 65	Yes: 2; No: 31	Yes: 1; No: 34
Prior Trauma History	Yes: 21; No: 47	Yes: 11; No: 22	Yes: 10; No: 25
Average Causality Score^d^	4.22/6	3.97/6	4.46/6

a Theravāda group includes participants from a follow-up study of practitioners of *vipassanā* according to the tradition of S.N. Goenka.

b For all data reported here concerning the onset of meditation-related-challenges, this refers to what practitioners took to be the *first* main period of challenges. Therefore, for the DLI sub-group, this cannot be taken as an indication of the first onset of DLI specifically, as for some subjects DLI may not have occurred until a later onset. Age at onset was not systematically queried. If the information was not volunteered during the course of the interview, it was calculated based upon participant age at time of interview, the year of interview, and the year of onset, when known. Data is missing for 2 subjects.

c Regular meditation practice was defined as once per week or more. Data is missing for 1 subject.

d Causality assessment in the VCE study employed 11 criteria, of which six are reported here. As described previously: “Six criteria were assessed as part of the demographics and attributes follow-up questionnaire or, in the case of non-responders, extracted from the interview transcript: (1) causal attribution to meditation by the subject (subjective attribution); (2) temporal proximity to (either during or following) meditation practice (challenge); (3) exacerbation of pre-existing symptoms following meditation; (4) occurrence on more than one occasion (consistency); (5) decrease when practice is reduced (de-challenge); (6) re-appearance when practice is repeated (re-challenge). A causality score was calculated as the sum of endorsements of these six criteria. Using standard guidelines, a score of two or greater, signifying ‘possibly related,’ was the cutoff for inclusion.” (Lindahl et al., 2017, p. 10).

**Table 2:** Participant demographics: meditation experts.

	VCE Experts
Total Number	33
Gender	Male: 25, Female: 8
Primary Type of Expertise	Theravāda: 14^a^
	Zen: 8
	Tibetan: 6
	Clinical: 5

a Theravāda group includes participants from a follow-up study of practitioners of *vipassanā* according to the tradition of S.N. Goenka.

## Results

Meditation practitioners who reported DLI did not differ from those who did not in terms of gender, age, race, ethnicity, practice tradition, practice intensity (retreat vs. daily), age of onset, having a meditation community at onset, working with a meditation teacher at onset, psychiatric history or trauma history (*p* > 0.05). The groups differed on the likelihood of being a meditation teacher: practitioners who were also meditation teachers were less likely to report DLIs (*p* < 0.05). The groups also differed on their amount of regular meditation practice prior to the onset of their challenges (*p* = 0.05). The DLI sub-group had more practitioners with more than 10 years of regular meditation practice.

### Typology of meditation-related DLI identified among VCE study participants

Descriptions of each of type of delusion-like ideation and examples from meditation practitioners are presented in Table 3.

**Table 3:** Types of meditation-related delusion-like ideation.

Type of DLI	Description	Example 1	Example 2
Being Controlled	Irrational, delusional, or paranormal thoughts about one’s body, actions, or thoughts being directed by an external force or agent, whether human or non-human.	*I still had this energy in me which was very wild. And probably its signature feature was that it felt like it was coming outside of what I had previously considered my own self. It had a will of its own and it was very powerful and willful and wanted things all the time, whether it wanted me to move in certain ways or to sound in certain ways.* [#30]	*I felt very connected with my body then. But the more internal I got, like with that paranoia, the more I felt like my body was kind of being controlled, and I started to make interpretations about who was controlling that or why.* [#57]
Death or Dying	Irrational, delusional, or paranormal thoughts concerning the impending or recent death of oneself or others.	*And then that night, when I lay down in my bed, I looked at my body and had this same sort of feeling that I was dead, even though I… Well, first of all I knew I wasn’t dead, and second of all it’s not like my body looked like it was decayed or anything. It looked exactly as it normally does. But I just had this feeling that I was dead, and it was sort of unshakeable. I could not convince myself.* [#84]	*There was a time when I thought, for some reason, that my teacher was passing away. And I ran over to his house on the monastery grounds. I ran over, ran inside, and basically he was sleeping, and I disturbed him. And it was basically not a good situation right there*. [#43]
Grandiosity	Irrational, delusional, or paranormal thoughts centered on conceiving of oneself as having an important or extraordinary role, mission, identity or ability.	*I started also getting visions about basically being sacrificed—these kind of messianic visions. The general sense was that I would need to sacrifice myself in some way for the benefit of saving mankind, or something.* [#85]	*I certainly had one of those experiences of having this sense in retreat of: I knew exactly what I needed to do with my job, and if I did this and then this would happen and then this would happen. You know, this whole grandiose idea that I was sure was this wonderful insight. And, the moment retreat was over, it was like, “What was I thinking?” I mean, it was just so totally off what would have really worked in reality.* [#62]
Ideas of Reference	Irrational, delusional, or paranormal thoughts interpreting ordinary or everyday events as being of special personal significance.	*I was convinced that that number was some sort of message to me about this whole experience I’d had during this retreat. […] And stuff came up on Google about the meaning of this number. And the number had some sort of spiritual or religious significance. […] But, somehow I interpreted this number as sort of heralding my enlightenment.* [#37]	*And then in his talk in the evening, Goenka mentioned something about a tree, and I understood that to be a message specifically to me to tell me that I needed to go sit underneath this tree and experience the suffering just as the Buddha did.* [#85]
Misidentification	Irrational, delusional, or paranormal thoughts about the identity of another person in which the other person is viewed as an imposter, a different person or entity, or simply not who they claim to be.	*And the reason I threw the water into my teacher’s face is I thought she was in cahoots with them and similarly with the guy who was the head of the center. The reason why I lightly slapped his face and the reason why I jumped out of the car was because, in my mind, I was in contact with someone who was trying to help me get away from all these people who were busy with this black Tantric plot, which I was trying to save the world from.* [#35]	*It was like this delusional atmosphere. Because I did not have thoughts myself anymore, it seemed. I was thought-less. It seemed like when I saw people, it seemed to me as if I knew these people already. I mean, I knew that I did not know them.* [#78]
Paranoia: General	General or non-specified irrational concern about or suspicion of people, places, or other phenomena beyond what is warranted or justified.	*I remember actually in the hour of meditation before I went to sleep, I kind of thought there was someone in my room. I was a little bit afraid. And then after the second wave of fear, I really just freaked out, I thought something terrible was going to happen.* [#63]	*But every sound is scaring me because every sound is the sound of something bad about to happen, like a crash in the zendo. And I’ve never been that scared in my life.* [#75]
Paranoia: Danger from Others	Irrational, delusional, or paranormal thoughts that have as their focus a concern about harm, persecution, or threat to one’s well-being and are attributed to the perceived or imagined actions of specific others, whether within or beyond the social world.	*I had like extreme paranoia. I thought I was being brainwashed. […] There were two periods where I was like, “I gotta get the fuck out of here. I’m going to become a zombie. I’m going to completely lose my ego. Like, I’m gonna just turn into a vegetable. I gotta get out of here. They’re trying to steal my brain.”* [#51]	*I thought I was reaching a higher state of consciousness, but at the same time I was very worried about these people from another place in the state—worried that they were there with malicious intentions and things like that. So, at the same time that I was getting high, I was very paranoid and very scared.* [#43]
Special Knowledge	Irrational, delusional, or paranormal thoughts concerning special knowledge obtained through extraordinary insight, experience, or interactions.	*I had instruments to play […]and I could not play these damned instruments for the life of me, and all of a sudden I’m chanting and the instruments are playing and there’s this deep voice coming out of me. It’s like I’m being shown how to do this practice by someone who has come in. I feel like I’m inhabited by a teacher, and they have come to show me, like I’m being taught.* [#27]	*I became somewhat clairvoyant in the sense that I could feel what was happening to other people in other locations. You know, only, at that time primarily people who were close to me.* [#48]

### Relative prevalence of different types of delusion-like ideation among VCE study participants

**Figure 1:**
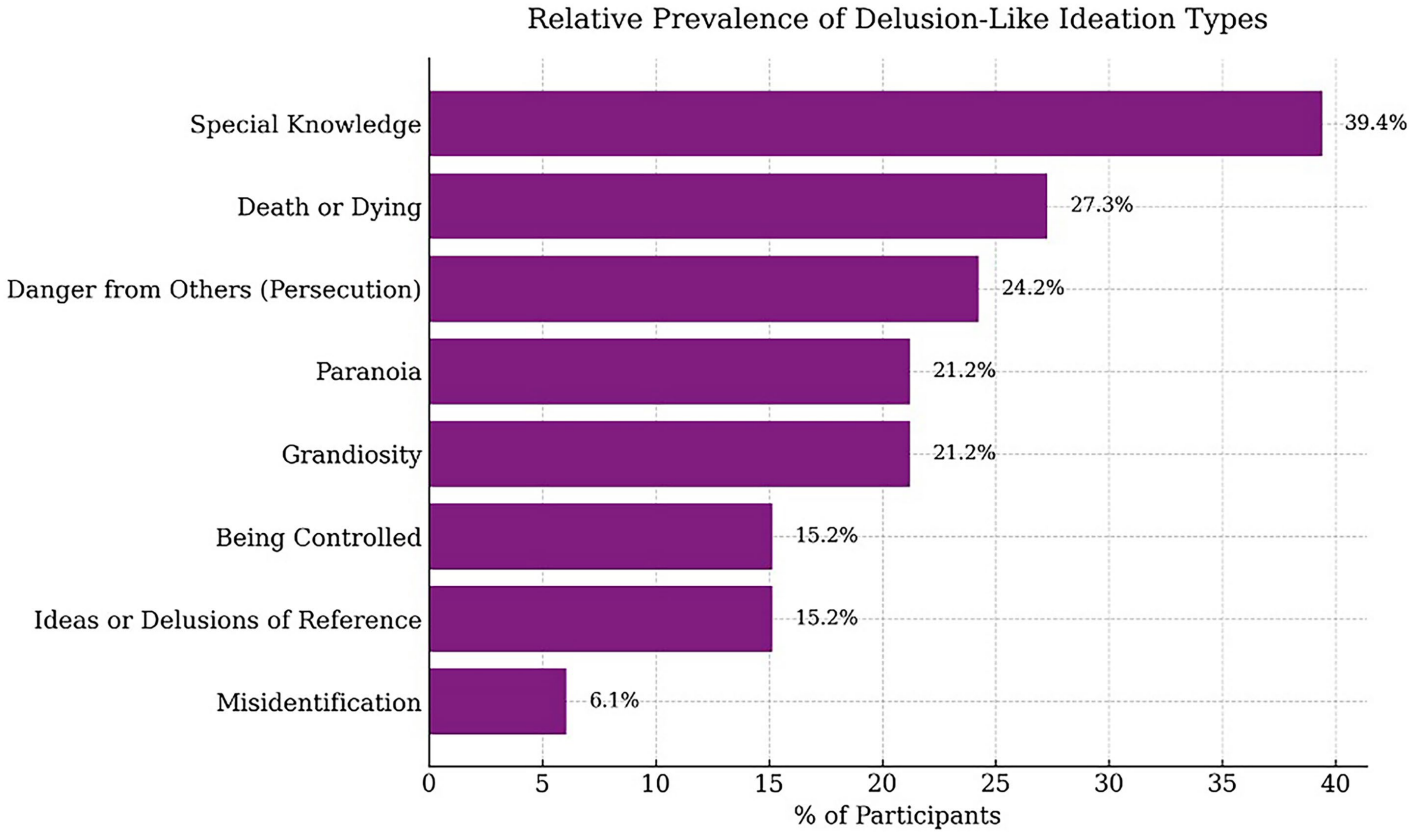
Relative prevalence of each type of DLI reported by VCE meditation practitioners in the DLI sub-group.

**Figure 2:**
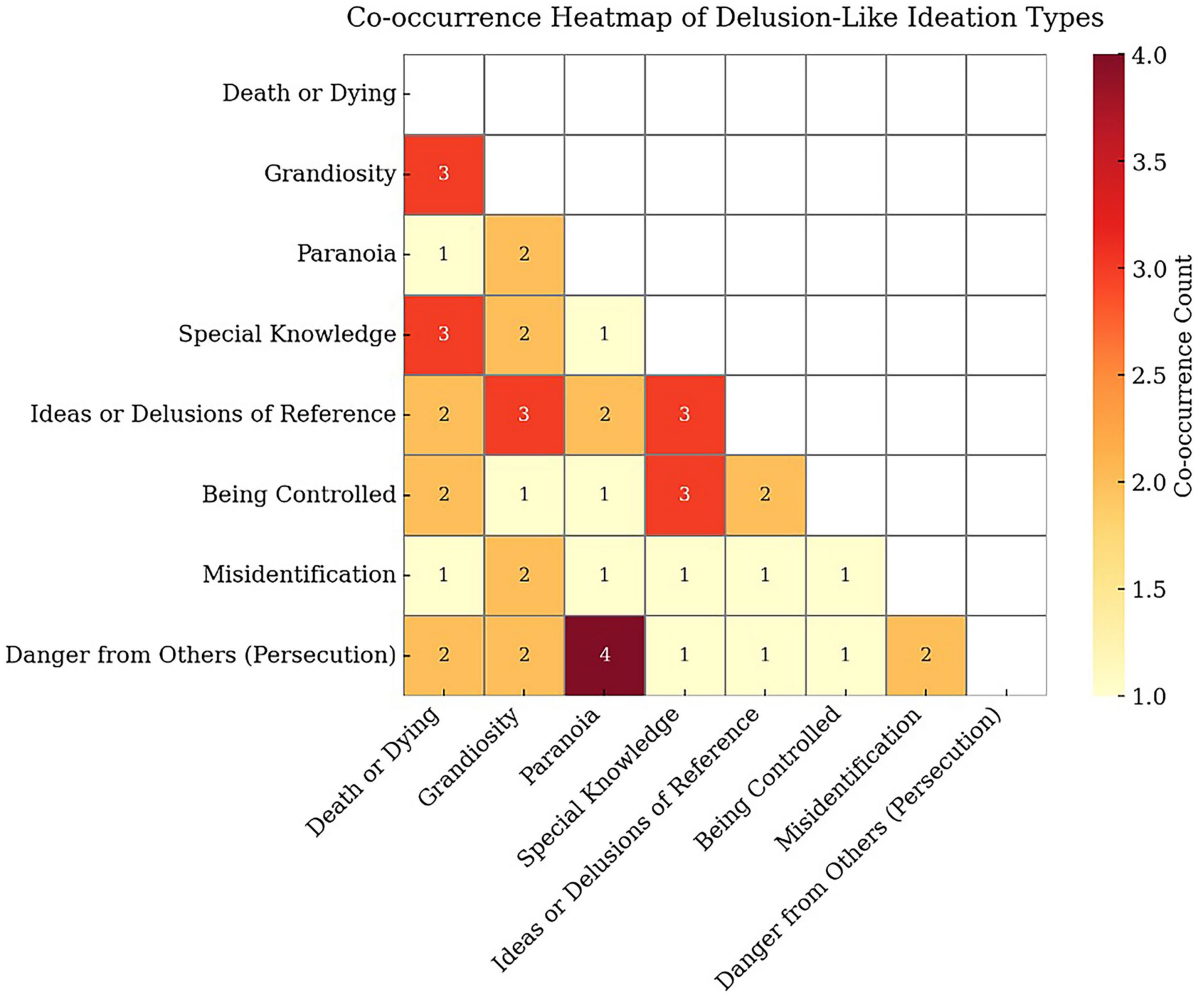
Co-occurrence matrix of pairs of delusion-like ideation.

Thirty-three VCE study participants (49%) reported one or more types of meditation-related DLI. The most prevalent type of DLI was Special Knowledge (13 of 33 participants, 39%), followed by Grandiosity (7, 21%); Death or Dying (9, 27%); Paranoia: General (7, 21%) and Paranoia: Danger from Others (8, 24%). Less commonly reported were Being Controlled (5, 15%); Ideas of Reference (5, 15%); and Misidentification (2, 6%). Miscellaneous references to an “Other” DLI not meeting criteria for our eight main types were reported by seven participants (21%). Participants in the DLI sub-group reported most commonly reported one type of DLI (16/33, 48%), with an average of two DLIs, and a maximum of six DLIs in a single participant. The most commonly co-occurring DLI were Grandiosity with Paranoia: Danger from Others (four participants); Special Knowledge with Being Controlled; Special Knowledge with Ideas of Reference; Grandiosity with Ideas of Reference; Death or Dying with Special Knowledge; and Death or Dying with Grandiosity (three participants for each pair of themes). See Figure 1 for distribution of types of DLIs. The frequency of types of DLI reflects information that was spontaneously volunteered by participants and may not reflect actual frequency of occurrence (Figure 2).

### Impacts and responses associated with meditation-related DLI

**Figure 3:**
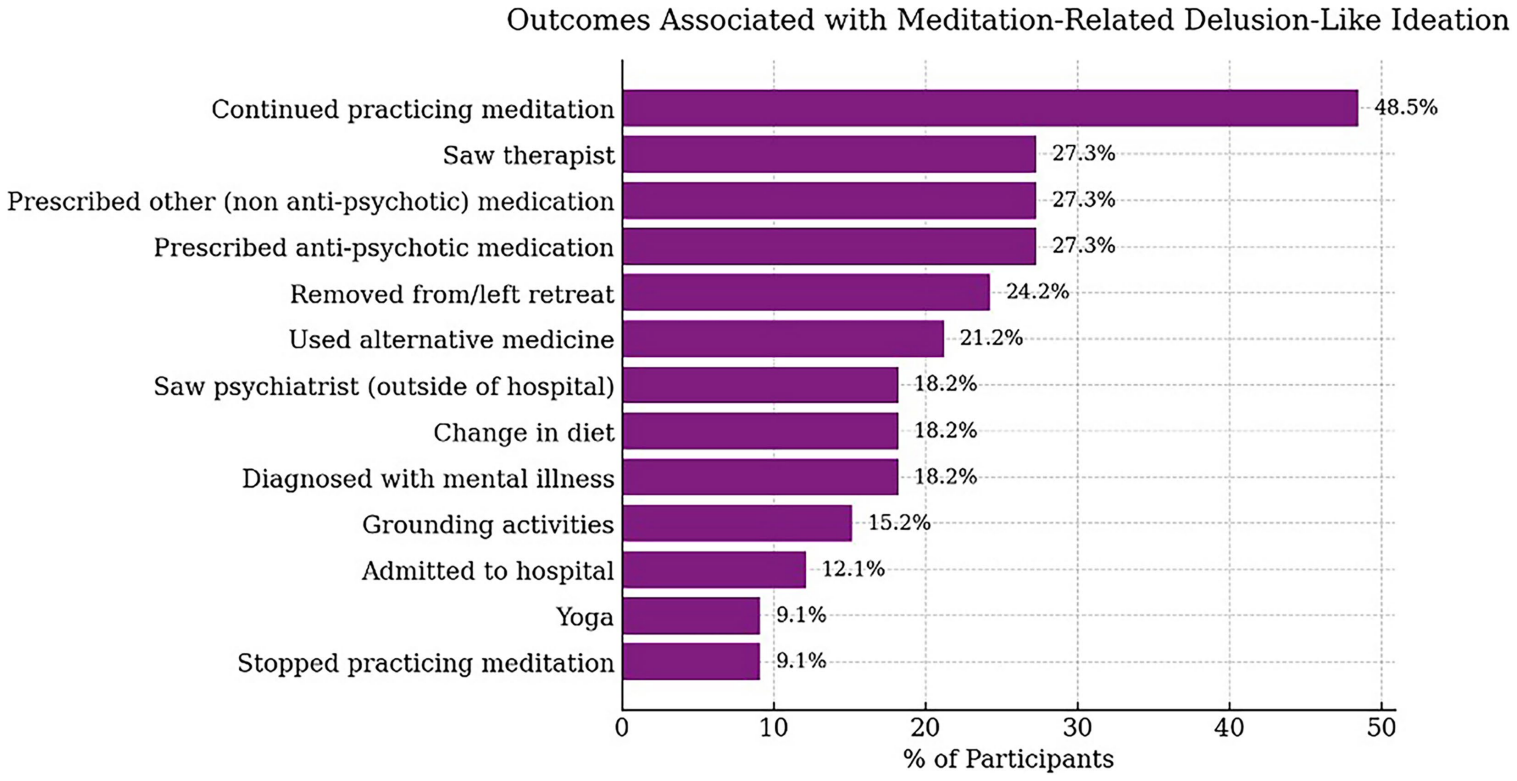
Range and relative frequency of outcomes associated with meditation-related DLI reported by VCE practitioners in the DLI sub-group.

Twenty-six participants in the DLI sub-group (78%) reported at least one impact or treatment response as a result of their challenging DLI. Sixteen meditation practitioners (48.5%) continued practicing meditation; nine (27.3%) saw a therapist; nine (27.3%) were prescribed non-anti-psychotic medication; nine (27.3%) were prescribed anti-psychotic medication; eight (24.2%) were removed from or left the retreat; seven (21.2%) used alternative medicine/holistic treatment; six (18.2%) were diagnosed with a mental illness; six (18.2%) saw a psychiatrist; six (18.2%) changed their diet; five (15.2%) used grounding activities; four (12.1%) were admitted to a hospital; three (9.1%) used yoga; and three (9.1%) stopped practicing meditation. These results are plotted in Figure 3. Because these data were based upon spontaneous reports, we cannot assume, for instance, that the participants who did not mention a therapist or psychiatrist did not consult one.

### Case studies of delusion-like ideation and their relationship to psychopathology

Meditation practitioners in the VCE study reported cases of delusion-like ideation that ranged from relatively transient and mild to acute and severe episodes resembling psychosis. Practitioners also described various trajectories associated with medtiation-related DLI. The duration, distress, degree of impairment, need for intervention, and type of intervention showed considerable variation, from transient and insignificant to enduring and requiring multiple kinds of intervention. In a minority of cases (6/33, 18%) DLI was limited to the immediate context of meditation and was addressed without either psychiatric diagnosis or associated treatments. Transient DLI was in some instances dismissed as a passing phenomenon associated with “retreat mind” (unusual experiences that may be expected to occur in meditation retreats), whereas others described transient experiences in exclusively psychological or psychiatric terms such as paranoia, irrational fear, delusion or delusional. In the vast majority of cases (27/33, 82%), however, DLI persisted outside of the period of formal meditation practice, and typically (22/33, 67%) were associated with distress and/or distruptive impacts on behavior and functioning.

Instances of meditation-related DLI were given a range of appraisals from practitioners themselves, from their teachers, from psychiatrists, and from other concerned people such as members of their meditation community and family members. Appraisals were also subject to change over time, with practitioners reporting how they held one view during, or in close proximity to, the episode but changed their appraisal after subsequent discussion with teachers, psychiatrists, or others. While seven practitioners (21%) held that their experience of DLI was psychiatric in nature, 15 (46%) received psychiatric care or were given a psychiatric diagnosis but nevertheless believed that their experiences were in part or entirely religious in nature. Eleven practitioners (33%) either never engaged with a psychological or psychiatric framework or rejected those interpretations. Appraisals of DLI were not determined solely by the duration or severity of the experience. That is, it was not the case that only mild, transient cases of DLI were appraised as normative, religious/spiritual, or paranormal while only enduring, distressing, and impairing cases were appraised as psychopathological. Some transient and even a few enduring and distressing experiences were appraised as wholly paranormal, as having religious significance, or as normative within the meditation community. This was particularly true for DLI that communicated “special knowledge” to the practitioner.

In the following section, we present four brief case studies of meditation-related DLI, illustrating differences in practitioners’ psychiatric, trauma, and practice history, as well differences in the types of DLI, their trajectory and impacts, and associated appraisals.

#### Case 1: delusion-like ideation in a meditation practitioner with a pre-existing condition of psychiatric illness

A 48-year-old male practitioner from the United States had a pre-existing history of mental illness, including a depressive episode at age 10 and suicide attempts at ages 19 and 23. Before the onset of his meditation-related challenges, he had been diagnosed with bipolar disorder and had begun taking lithium for a year at his parents’ insistence. At around the same time, while in college, he began studying and practicing Zen and proceeded to interview three prominent Zen teachers in the United States in search of a good fit. He recounted that one of the Sōtō Zen teachers he ended up practicing under “did not at first believe” how easily he had some anomalous perceptual experiences during meditation. After a year, he went off lithium. During this period, the intensity of his studies and the intensity with which he approached his meditation practice resulted in numerous periods of transient mania or hypomania. He explained how “with the mania there came a heightened ability to focus, and so I became hyper-focused in my Zen practice,” which was a sound-themed *kōan* (a contemplative prompt in Zen Buddhism that motivates inquiry into sound and hearing) that he received from his teacher. He described how at one point he “acquired the delusion that [teacher’s name] had chosen me as his successor—and this was just a manic delusion.” A couple of times during this period he went to his teacher’s center to practice for the day, and on one of those occasions, while he sat meditating with the group:

the whole thing suddenly became absurd to me. […] The bell had rung to end the sitting meditation and to begin the walking meditation, and I was in such a deep meditation I did not want to interrupt it, and I did not move. And so one of the nuns came up to me to prompt me to go out and start walking meditation, and I roared at her—like a lion’s roar. And this was just mania. And I was immediately moved [to] a second overflow meditation hall. I’m amazed that they did not kick me out at that moment.

While in this second meditation hall, he “started thinking about the situation as, in a way, being a giant club with everybody having their robes and it being just more of a social milieu than a training center.” This soon led to an outburst of “hysterical laughing” at which point “one of the monks came in and said, ‘[practitioner’s name], this is not Zen. You have to leave.’ And he walked me to the car.” He was invited to return to the monastery only when he was stable and with a note from a psychiatrist deeming him fit to continue meditation training. He did not contact the Zen center again for 25 years. Looking back on his earlier period of Zen practice, he characterized some of the experiences as being “all delusional stuff.” However, other concurrent changes in his sense of self, especially changes to his sense of self-world boundaries, seemed to him more in accord with meditative attainments described as *satori*. He said, “it’s hard for me to be clear on whether the illness was distorting the experience or whether this was genuinely a *satori* experience, but I had never experienced anything like this in mania before.” After stopping meditation for long periods during his 25 years away from the Zen center, he has since resumed a gentler practice in more recent years, with no reported ill effects.

#### Case 2: delusion-like ideation emerging as a new condition in a meditation practitioner without a prior psychiatric diagnosis and resulting in a new diagnosis

A 36-year-old female practitioner from Eastern Europe with no history of mental illness described how her initial encounter with meditation led to the diagnosis of a psychiatric disorder. She had experienced a challenging family life that included sexual abuse. She did not begin meditating until she was 32, when she attended a 10-day retreat on the theme of emptiness led by a Tibetan Buddhist teacher in the Gelug lineage. About 5 days into the retreat, she says, “I entered in a kind of trance” and “achieved the point where I had no thoughts for about 3 days.” Then, she had what she described as a dissolution experience that led her to feeling scared that she might die or leave her body. She also “started to begin to think that there was something special with me, and that the teacher has something special to tell me.” At the same time, she remained fearful and paranoid. On account of these experiences, she went to the main teacher and asked him, “I am going crazy or something?” He replied that she should continue to meditate on emptiness with the intention of being of benefit to all people. She did not find this particularly helpful and also asked a supporting teacher if progressing in meditation is supposed to be “so frightening.” He replied that “it should not be like this. It should be a reason to be happy, not to be afraid.” Her boyfriend, who was also attending the retreat, suggested that she stop practicing, but she did not.

On her way home from the retreat, her “paranoia and distortional thoughts amplified.” Things around her, such as the traffic signs on the side of the road, began to have “special content,” where “everything around me was addressed to me.” At this time, she also thought her friends “were some bad persons who were trying to hurt me.” Three days later, as her condition grew worse, she was hospitalized. She was given two injections of diazepam, and after 16 hours of sleep, she began to feel better. Three weeks later, however, she resumed practicing meditation because she felt a “special need inside to restart that experience.” She “did not expect to go psychotic again,” but after about 3 days the “distortion of thoughts, anxiety, paranoia appeared again.” She also experienced “distortional thinking, about hell, about evil, about demons,” which she characterized as a “wrong interpretation of reality.” This included believing that people around her were demons. Ultimately, she was stabilized when a doctor came to her home and gave her injections of haloperidol; soon after, she was diagnosed with “polymorphic psychotic disorder.” Though her childhood traumas previously caused challenges such as nightmares, neither she nor her psychologist thought this was the cause of her psychosis; both understood the meditation retreat to have been the cause. Both her psychologist and her boyfriend said that prior to the retreat, she was “a normal person” without “psychiatric problems.”

#### Case 3: delusion-like ideation emerging as a new condition in a meditation practitioner without a prior psychiatric diagnosis but resolved without hospitalization or diagnosis

A 28-year-old male from the United States described growing up in a “warm and affluent” family. He had no history of trauma or mental illness prior to beginning meditation practice at age 21. At age 23, having had only positive experiences during three prior 10-day meditation retreats in the tradition of Vipassanā as taught by S.N. Goenka, he undertook his fourth course. On this retreat, he “went after it with a lot more energy and determination.” Around the eighth day of the retreat, he reported having “messianic visions” about the “need to sacrifice myself in some way for the benefit of saving mankind.” At first, he was thinking “‘Okay, this is kind of crazy.’ But then I lost the ability to realize that those things were just mental images. And so, I started having what I understand to be ideas of reference.” For instance, he described how in the evening pre-recorded discourses, “Goenka mentioned something about a tree, and I understood that to be a message specifically to me to tell me that I needed to go sit underneath this tree and experience suffering just as the Buddha did.”

He soon came to believe that the entire retreat was a “set-up” and that you “actually became free when you left.” One evening, he marched into the meditation hall, pointed at one of the teachers, and declared “I am!,” believing this to be the code word by which the teachers would recognize his insight into the “set-up.” Instead, one teacher suggested he go to the hospital and had an assistant drive him there. However, the assistant did not insist he go in, leaving the decision up to him. He thought this was another part of the “set-up” and declined to enter the hospital. Following this, he resumed the retreat. He was even allowed to drive other retreatants home, but the other retreatants ended up asking to be let out of car after becoming unnerved by his behavior. Back home, he continued his “narrative” of enlightenment by sitting underneath a tree in the lotus posture, with his “terrified” parents finding him there and insisting he get help. For the next few months, he met with therapists but never received a psychiatric diagnosis or took any meditations. He also met with meditation teachers and believed that there were spiritual aspects to his experience as well as delusional aspects, but he failed to find anyone to validate his experiences one way or the other. His “ideas of reference” gradually went away on their own over the course of 6 months.

#### Case 4: delusion-like ideation emerging as a new condition in a meditation practitioner without a prior psychiatric diagnosis, not resulting in intervention, and appraised as spiritual or paranormal

A 51-year-old male from the United States with no history of mental illness grew up in a challenging family environment with “low-grade trauma.” He began practicing Vipassanā “on and off” at age 25 and then increased the regularity and intensity of practice at age 35 following a personal crisis. After about 6 months, he began to experience challenges arising from meditation, primarily what he described as energy-like somatic experiences and involuntary movements. On account of this “energy” he would find himself “thrust into different yoga poses, some of which I knew, some of which I did not know,” including into postures that “there was no way my current physical condition could hold.” Over the course of many years, he developed a relationship with this “energy” as a guide or spiritual teacher, while at times also experiencing it as a “trickster” pushing him towards unsafe or unusual actions. Because the presence and impact of the energy was often associated with healing and with insights, cultivating a relationship with the “energy” became central to his spiritual path, and he ended up dropping Vipassanā as his primary practice. From this point forward, his main orientation has been “discernment” regarding how to “surrender” to an energy that he understands as having its own will while still maintaining a healthy sense of boundaries and self-agency. He believes the energy is regularly “testing” him and “showing me my edges.” Illustrating this, he recounted a time when

I was in a bath after a basketball game, and I felt the energy arising and it lifted me up out of the bathtub, and I started walking over to the sliding glass door on the second floor where I was, and I felt this energy surging in me—like an energy that wanted to lift up and fly. And I was totally aware but I was letting the energy move me, and I opened the sliding glass door, and I kept watching, even though I was a little bit nervous. And then, suddenly, I saw and felt my leg was lifting up as if I was going to climb onto the edge of the balcony. And I understood in this moment—just like in a flash—that, yes, this energy *did* want to fly, it didn’t know that I couldn’t fly, and that if I actually let my next leg go up on the balcony, I would crouch and take off and then probably crash on the cement below and die! […] So with a lot of fear and concern, I pulled back and I was trembling and I realized: ‘Okay, wait—I *can’t* always trust this energy.’ […] I had to navigate when to step in and when to step back.

Rooted in these ongoing experiences, he has become a spiritual teacher, and he understands the energy as often working through him to heal and teach others. Ultimately, he said, beneath the “strange phenomena and the wildness of it all,” the experience has been one of “heart opening” and “peace” that has “changed everything,” “anchoring” him in “loving-kindness” and enabling him “to be the teacher that I am.” Though he has had guidance from others (mostly healers and spiritual teachers) in navigating his challenges, he has never sought out psychiatric help or understood his experiences through that lens.

### Experts’ views on meditation-related DLI

Of the 33 meditation experts interviewed in the VCE study, 10 (30%) reported one or more types of DLI as a kind of meditation-related challenge they had encountered among their students or clients. Like the meditation practitioners interviewed, special knowledge was most commonly reported by experts (6/10, 60%), followed by grandiosity (4/10, 40%) and general paranoia or danger from others (4/10, 40%). Most of the references to DLI among the experts were taken as concerning indications that close monitoring or immediate intervention was warranted. Half of the experts who commented on DLI (5/10) had dual training as meditation instructors and as clinical psychologists or medical doctors. One stated that, in his experience, if a practitioner is “hearing voices telling you spiritual things […] or if you start experiencing yourself as a deity, you are probably gonna get directed away from that.” Multiple experts also noted that it was possible for practitioners to have unusual experiences that led them to conclude “Oh my God, I’m enlightened,” or to “think they have special powers,” or to “think I’m special, and I was reincarnated from a particular planet.” This would cause some experts to ask: “Is this something [indicating] that this person’s heading into trouble?” Two experts also specifically noted a potential concern for students who believed about their teachers “‘[I] feel like you are reading my mind’” or “‘I know what you are thinking of me.’” These beliefs about mind-reading or mental control could become associated with paranoia and even hostility towards the teacher.

Three experts, however, normalized certain meditation-related DLIs. One Tibetan Buddhist teacher explained that certain meditation experiences (Tib. *nyams*) could involve intense paranoia as well as becoming “totally lost and totally involved by the experience and thinking that it’s real.” One Theravāda Buddhist teacher associated beliefs in being enlightened or having special powers with the well-known “corruptions of insight” (Pali. *vipassanā-upakkilesa*) in his tradition. Finally, another Theravāda Buddhist teacher indicated that unusual insight into the sense of self could be normative: “if you were in the grocery store and somebody said this to you, you might think, ‘This is really a problem.’ But on retreat, in this context, we do not see it that way.”

## Discussion

Reports of delusion-like ideation among Western Buddhist meditation practitioners in the VCE study fall into eight thematic categories, with special knowledge, death or dying, grandiosity, and paranoia being the most prevalent. More than half of our participants endorsed two or more types of DLI. Despite these challenging experiences, many participants continued practicing meditation and did not report being diagnosed with a psychiatric disorder. Others were instructed to discontinue practice and receive professional psychiatric attention, and some were diagnosed with an emergent mental illness, prescribed psychotropic medication, or hospitalized.

The content of these DLIs, as well as the response to them, often closely reflected the existential aspects and contextual factors of meditation culture. By situating meditation-related DLI in the context of lived experiences, patterned by local cognitive and cultural practices, we argue that our findings support a socio-biological view of the development of delusions and the spectrum of psychotic disorders.

### Delusion-like ideation reported by Buddhist meditation practitioners in the West

Among the meditation practitioners interviewed in the *Varieties of Contemplative Experience* study, nearly half the sample reported at least one instance of DLI associated with their meditation practice. No significant demographic differences emerged when comparing VCE study participants reporting DLI and those not reporting DLI. Both groups had similar distributions of gender, race, and ethnicity, and they did not differ in their age of onset of their first meditation-related challenges. DLI was reported among practitioners of different Buddhist lineages in roughly equal amounts. Those reporting DLI were just as likely to be practicing in a meditation community and working with a meditation teacher. DLI could result from a range of different practice intensities, including retreat, daily practice, or a combination. This expands the scope of previous case studies that had associated meditation-related DLI largely with intensive meditation retreats. Importantly, psychiatric and trauma histories were not more common in the DLI group. Practitioners were also not more or less likely to attribute their challenges to meditation if their experiences included DLI. Overall, the between-group similarities suggest that meditation-related DLI cannot simply be attributed to a pre-existing mental illness or to certain practice contexts (whether intensity, type, or tradition of meditation practice).

There were two between-group differences. One was that meditation practitioners who identified as meditation teachers were less likely to report DLI. This could indicate that the degree of expertise associated with being a meditation teacher made DLI less common or less challenging. However, this is undermined by the finding that the DLI sub-group contained more practitioners with more than 10 years regular meditation practice prior to the onset of challenges. Another possibility is that meditation practitioners who reported DLI were less likely to have been or later become meditation teachers or that meditation teachers were more likely to refrain from disclosing DLIs that they had experienced.

The four case studies chosen to illustrate the impacts, trajectories, appraisals, and context of meditators reporting DLI show multiple patterns. Only one type (for a total of 2/33 practitioners reporting DLI) falls into the pattern of a prior psychiatric diagnosis being exacerbated by meditation. While experiences with DLI could be sufficiently severe, impairing, and concerning that they warranted hospitalization and a diagnosis of an acute or transient psychotic disorder, this was not universally the case either. Delusion-like ideation could manifest in transient forms that, while concerning and impactful for a time, were resolved without diagnosis or medication. And some instances of DLI were never appraised in psychiatric terms at all. The norms and worldviews of Buddhist meditation traditions also provided resources for normalizing certain DLI, even those that were thematically similar to experiences that were pathologized. In a few cases, practitioners or experts came to view certain DLI as expected on retreat or even indications of a kind of progress.

The experts interviewed in this study sometimes drew upon Buddhist concepts in normalizing certain DLI. This is in accord with Buddhist textual sources that both recognize the possibility of DLI emerging in the context of meditation, while also cautioning that such experiences can be both unpleasant for practitioners and lead them to erroneous self-views (Lingpa, 2015; The Śūraṅgama Sūtra, 1966). However, the presence of DLI led to significant concerns about the practitioner, especially if the DLI was persistent and associated with unusual (or aggressive) behavior.

In line with previous research that has proposed that culture influences delusional content and potentially even frequency of DLI categories (Gold and Gold, 2025), this analysis of DLI among Buddhist meditation practitioners in the West identifies a unique set of DLI themes. Taken together, paranoia (both general mistrust and feeling of danger from others), special knowledge and grandiosity, and death and dying, were the most commonly reported in this sample. This is largely consistent with findings in other communities, where persecution and grandiosity are often reported as the most common delusional experiences both in patient populations (Grunfeld et al., 2023; Pappa et al., 2025; Picardi et al., 2018) and in general healthy individuals (Freeman, 2006). Among this sample, grandiosity commonly manifested in ways related to the meditation practice context, with practitioners coming to believe that they were enlightened, a Buddha or a messianic figure, or involved in a supernatural struggle between forces of good and evil. Similarly, special knowledge, often understood in the clinical literature as a subtype of grandiosity, may also originate in the idea that the practice of Buddhist meditation is a knowledge-seeking and an expertise-building enterprise. While some instances of special knowledge were dismissed as inauthentic deviations from the genuine path, others were more ambiguous when considered in the light of Buddhist worldviews and cosmologies, which include supernormal mental powers, other realms of existence, metempsychosis, and non-human, non-physical entities such as spirits, gods, and demons.

Lastly, DLI focusing on themes of death and dying were relatively common in our sample (27% of participants reported this type of the DLI). In clinical populations, delusions of death or dying, classically known as the Cotard delusion, are extremely infrequent in psychotic patients (<1% prevalence) (Bott et al., 2016; Dihingia et al., 2023), and are often associated with such conditions as dementia (Cipriani et al., 2019) or psychotic depression (Faunce and Tennant, 2024). Even though the death and dying themed DLI reported by our participants rarely resemble the Cotard delusion, the fact that this very uncommon DLI theme is reported so frequently in our sample may reflect how Buddhist meditation culture emphasizes contemplating mortality in particular and the purpose of existence in relation to living and dying more generally.

Perhaps more DLI would have been framed within the religious cosmology and soteriology of Buddhism had they taken place in another cultural context. However, given that Buddhist meditation in the West has often been presented in psychological terms in which ritual and cosmology are downplayed (McMahan, 2008), many instances of DLI were evaluated this way by practitioners and teachers alike. Nevertheless, it is worth again recalling that Buddhist literature has long acknowledged that DLI such as grandiosity and paranoia can occur as a result of meditation (Lingpa, 2015; Salguero, 2023; Sayadaw, 1965; The Śūraṅgama Sūtra, 1966) and are in this literature commonly evaluated as deviations or abnormalities.

### “Retreat mind”: the potential roles of sensory deprivation, attenuation and enhancement in the onset of delusion-like ideation

The physiological and environmental conditions of intensive Buddhist meditation may be of special interest in understanding meditation-related DLI. Intensive meditation practice is often carried out in contexts that facilitate unusual experiences through prolonged periods of immobility, isolation or sensory deprivation, and social isolation (Lindahl et al., 2014). Furthermore, chanting, single-pointed concentration (contemplative practice focused on a single object of experience, such as breath, sound or mental image), and other attentional practices may result in altered or novel perceptual and cognitive experiences. Indeed, meditation experiences have long been referred to as “altered states of consciousness” (Shapiro and Giber, 1978). According to the “experiential source hypothesis” (Hufford, 1989), lived and shared experience of ambiguous and strange experiences may give rise to shared local and folk phenomena, including spiritual, religious or paranormal beliefs.

Sensory deprivation occurs relatively rarely in normal circumstances, and is known to produce a variety of experiences, including delusions, perceptual distortions, and hallucinations (Mason and Brady, 2009). Meditation retreats are often designed to remove and reduce typical environmental cues, to alter social and occupational contexts, and to change biological rhythms (early waking, early bedtime, fewer mealtimes or meals taken at atypical times of the day). These changes can be powerful in situating the retreat participant in temporal and spatial contexts that are a clear break from the lay world and are indicative of a different kind of activity. Meditation retreats often involve dramatic changes in environment, schedule and behaviors, a reduction in sleep, and restricted social activity or none at all. In addition, the practice of meditation itself is designed to attenuate sensory inputs by focusing attention on a pre-selected environmental or physiological sensation or away from the perceptual world altogether upon a specific thought, concept, or mental image. It should not be surprising that meditation retreats with such features have been found to be associated with altered states of consciousness (Galante et al., 2024), including changes to the sense of self and to the sense of body boundaries (Ciaunica, 2024). Social deprivation in particular has been associated with a significant degree of mental distress leading to hallucinations, sensory-perceptual alterations, and a potential for the development of psychotic disorders (Arrigo and Bullock, 2008; Smith, 2006). Even the social isolation brought about by the COVID-19 pandemic contributed to increased psychotic symptoms (Alle and Berntsen, 2021).

Changes in sensory experiences in retreats and during intensive meditation practice can range from different degrees of sensory attenuation or deprivation (e.g., silence, immobility), to sensory enhancement (e.g., increased attention to physical sensations, sounds or mental imagery). Given that deprivation can lead to increased perceptual sensitivity (Lindahl et al., 2014), these two mechanisms might also be related. These sensory changes associated with intensive meditation practice or certain meditation retreat environments, may shed light on some of the underlying psychophysiological mechanisms of anomalous meditation-related challenges, including DLI.

### The spectrum of psychosis

The question of whether there could be a relationship between contemplative practices and psychosis appears tendentious because the concept of psychosis, both inside and outside of psychiatry, takes schizophrenia and bipolar disorders as the sole exemplars, with the understanding that severe mental disorders are also biological illnesses. Recent views, however, suggest that psychosis is undoubtedly a family of brain disorders, and, in the case of schizophrenia and bipolar disorder, the causes include genetic ones. However, there has been evidence for many decades that some psychoses are associated with social risk factors–in particular, adverse life events, especially in childhood, being a first- or second-generation immigrant, and, in some parts of the world, urban living (Morgan et al., 2008; Radua et al., 2018). Not all psychosis is chronic; it can also be brief (Pillmann and Marneros, 2003). In DSM-5, this diagnosis is referred to as *brief psychotic disorder* and in ICD-11 as *acute and transient psychotic disorders* (Fusar-Poli et al., 2022). DSM-5 diagnostic criteria (American Psychiatric Association, 2022) include the presence of delusions, hallucinations, disorganized speech, or grossly disorganized or catatonic behavior which lasts more than 1 day but less than 1 month followed by a rapid return to normal functioning. One of the specifiers is the presence of a stressor; in this case, the episode is characterized as a *brief reactive psychosis.* Like other forms of psychosis, brief psychotic disorder is thought to be caused by both biological and social factors including cultural ones such as “conflicts between traditional and modern culture, migration, seeking refuge from one’s country of origin due to persecution, rapid social changes… [or] life events such as marriage or religious experiences” (Fusar-Poli et al., 2022:75–6).

Finally, not all psychotic episodes are part of an illness of any kind. Psychotic symptoms are now believed to lie on a continuum that includes both clinical and non-clinical states, and one can experience psychotic states without having, or developing, an illness. It is estimated that about 5% of the population will experience psychotic symptoms, in contrast to the less than 1% that will be diagnosed with schizophrenia (Charlson et al., 2018). Further, schizotypy traits and psychotic-like experiences, which include DLI, are highly prevalent and likely underestimated in healthy individuals. While the psychotic experiences of some of the individuals in the VCE study may represent the first signs of a chronic illness, for others their symptoms were non-clinical, transient, and potentially reflect an underlying cognitive response to their meditation-related or personally challenging experiences. That said, those symptoms still required treatment in some cases and were associated with important degree of distress. Fusar-Poli and colleagues (Fusar-Poli et al., 2022) note that “brief psychotic episodes have been historically difficult to accommodate as a so-called third psychosis in the dichotomy of schizophrenia and bipolar disorder” (p. 72). The existence of such episodes, however, is crucial for evaluating the nature of DLI in our sample. With respect to the meditation practitioners who are the focus of the study, even when DLI appear to come on suddenly, last a short time, and then remit completely, they may nevertheless count as symptoms of illness without the implication that they are pathognomonic of a psychotic disorder. Further, brief but recurrent psychotic states, when left untreated, can develop into more serious and enduring disorders (Howes et al., 2021).

### Meditation-related DLI as idioms of distress

Challenging experiences may be expected as part of a spiritual or religious path, and they may also be at odds with normative mental health frameworks (Lindahl et al., 2020). As demonstrated by their average causality scores, participants in the VCE study reported that their challenges, including DLI, were triggered either specifically by the meditation technique and/or the circumstances associated with intensive meditation practices (e.g., retreat settings, immobile postures, altered sleep–wake schedules, and social isolation, among others factors). Some meditation practitioners and meditation experts appraised DLI as a normal side-effect of meditation, whereas others viewed them as concerning and warranting intervention.

Culturally sanctioned responses and local idioms of distress have been taken into greater consideration in contemporary psychiatry. With respect to the diagnosis of psychotic disorder, DSM-5 specifies that one should “not include a symptom if it is a culturally sanctioned response.” The condition on the cultural inappropriateness of diagnosing an experience as delusional is in keeping with the DSM-IV condition on delusion (abandoned in DSM-5) as a belief that is held “despite what almost everyone else believes.” Thus, causal attributions and ascriptions of salience, meaning, or value depend not only on the resemblance of any given DLI to clinical definitions of delusions but also to the norms of contemplative practice traditions.

We have already noted that the content of some meditation-related DLI express ideas that can be found in a variety of contemplative traditions. For instance, one Tibetan Buddhist practitioner (see a brief vignette of her experience in Table 3: Special Knowledge) describes working with a teacher as encountered in the interior world of her own experience. Such encounters are described in Tibetan Buddhist literature (Lingpa, 2011, 2015) and can be valued as genuine signs of attainment. By virtue of these experiences being shared by, and legible to, a practitioner’s peer group, one might conclude that they cannot strictly be considered delusional. This is not sufficient, however, to define a DLI as culturally appropriate. In the case of this practitioner, the impact of this teacher on the direction of her practice was considered both by herself and by her teacher as invalid, potentially dangerous, and in need of course correction. Thus, even when a cultural framework is available for normalizing a DLI-related meditation experience, other considerations—in this case her own increasing destabilization and the opinion of her meditation teacher—were overruling factors in determining the significance of the DLI and the need for intervention.

Contemporary discourses about delusions are quite sensitive to cultural norms and tend to incorporate them. In recent years, there has been a growing awareness that delusions may be social cognitive phenomena that represent, and may be responsive to, the social or cultural environment (Bell et al., 2021). Current work on the intersection between psychiatry, philosophy and anthropology offers perspectives that may help to integrate social, biological, spiritual, contextual and cultural domains to understand how lived experiences are shaped and appraised. It has been proposed that the contexts the context and interpretation of experiences shapes the phenomenology of symptoms through a process of “social kindling” (Luhrmann et al., 2015). A related notion is that of cultural affordances (Kitayama et al., 2006; Ramstead et al., 2016), which refers to the idea that experience is not lived as neutral but is embedded in, and scaffolded by, the culturally learned possibilities for action, meaning, and response that are available in one’s sociocultural niche.

These dynamics are particularly clear among “culture-bound syndromes”–psychiatric disorders traditionally understood as restricted to a particular geographic region or culture (Ghosh and Chowdhury, 2020; Mianji and Semnani, 2015). Two forms of travel that can be construed as pilgrimage have been associated with brief psychotic experiences: “Stendahl syndrome” in which exposure to great art (classically in Florence) produces psychiatric symptoms including hallucinations (Palacios-Sanchez et al., 2018); and “Jerusalem syndrome”—a brief psychotic syndrome characterized by religious delusions in visitors to the holy city (Bar-el et al., 2000). A similar syndrome was recently documented following Muslim pilgrims’ visits to Mecca (Sahin et al., 2022). Jerusalem syndrome is a particularly apt analogy for the present case. Some individuals who have been characterized as suffering from Jerusalem syndrome appear to have been developing psychotic symptoms before arriving in Jerusalem and may have decided to travel there in response to delusional religious ideation. There is evidence, however, that a sub-group of patients show no signs of prior psychiatric disturbances or history. When they arrive in Jerusalem, they undergo a characteristic decompensation over a number of days during which religious delusions are central, and they tend to recover within a few days. Jerusalem is a profoundly meaningful place for several religious traditions, not least because it is a liminal space—a place of transition between the realms of quotidian life and the holy (Yeager, 2019). In some individuals, this encounter is a stressor that appears to play a role in the etiology of brief psychotic experiences. Contemplative practice has been theorized as a profoundly meaningful journey of psychological transformation (De Wit and Baird, 1991) and meditation retreats share some of the social features of traditional pilgrimages, such as separation from ordinary daily life, temporarily committing to atypical ascetic behaviors, and engaging in rituals, among others. To the extent that psychotic symptoms can be brought about by encounters with deeply meaningful and transformative places, objects, or practices, meditation and the cultural contexts of meditation retreats may act as a catalyst as well.

Similarities between culturally normative and pathological thoughts may be distinguishable to individuals within the cultural group when they cannot be distinguished by clinicians. As illustrated earlier in this section, the fact that some instances of DLI documented in the VCE study were identified by a practitioner’s meditation teacher as non-normative is significant evidence that some DLI will remain pathologized even in a religious context. The interviews with meditation experts who were often concerned with DLI as non-normative provides further supporting evidence. From this perspective, the DLI reported in our paper can be understood as idioms or cultural concepts of distress (Lewis-Fernández and Kirmayer, 2019; Nichter, 2010), specific not to a particular geographical location, but to a distributed community of Western Buddhist meditation practitioners, who share common concerns, practices, and aspirations. Idioms and cultural concepts of distress refer to context-specific modes of experiencing and describing psychological suffering and to locally accepted strategies for seeking help (e.g., deferring to a meditation teacher or asking for psychiatric intervention).

### How DLI experiences are appraised and when they require intervention

Many participants reporting DLI in our study continued practicing meditation, but some also sought professional help. Eleven practitioners were prescribed antipsychotic medication, and eight were diagnosed with a new mental illness. Five participants were admitted to a hospital as a result of their challenging meditation-related DLI experience. Many participants appraised their DLI in a mix of Buddhist and Western biomedical language, reflecting mixed and multiple contexts in which these experiences are understood. Since some of the DLI reported in this study reflect normative Buddhist worldviews, not all may be delusional. Still, a number of participants required psychiatric care as a result of their meditation-related challenges accompanied by DLI. Psychotic experiences with religious themes have been widely documented (Collin et al., 2023; Cook, 2015), are common in patients with schizophrenia, and were even suggested to be a marker of disease severity (Siddle et al., 2014). Thus, DLI with religious themes can have a variety of interpretations, ranging from normative religious experiences, to practice-related challenges, and to potential psychopathology.

As Kaselionyte and Gumley (2019) suggest, the interpretation of extreme mental challenges in the context of meditation experience presents a danger of falling into one of two narrow discursive frameworks: a normative biomedical one, focused on therapeutic solutions to mental health events, or a religious one, which may normalize states of distress. Rather than attempting to determine the “true” nature—psychiatric or religious—of these experiences, previous analyses of the VCE study data found that both meditation practitioners and meditation experts alike were often more concerned with determining when such challenges warrant additional support or intervention (Lindahl et al., 2020). These practical decisions were made based upon 11 criteria, most of which had been identified in earlier literature attempting to differentiate spiritual or mystical experiences from psychosis or mental illness. The 11 criteria for differential diagnosis or determining the need for intervention included (1) the primary, phenomenological qualities of the experience (i.e., what is the experience?); secondary associated qualities such as (2) distress; (3) functional impairment; (4) duration; (5) sense of control over the challenge; and (6) critical attitude towards the challenge; temporal features such as (7) positive or negative impact across time; and contextual features, including (8) the circumstances of onset; (9) health history or prior conditions; (10) compatibility with a meditator’s cultural background; and (11) the knowledge, skill and resources available to the meditation teacher. Across the various instances of DLI in the VCE data set, each of these criteria were applied. Critical attitude—sometimes called intact reality testing—was an important criterion associated with DLI and hallucinations in particular. The case studies also reveal that duration, degree of distress and functional impairment, behavioral changes, health history, and compatibility with cultural norms could lead to a DLI being appraised in pathological or religious terms. Investigating how these criteria are applied by meditation practitioners and meditation communities suggests that the presence of DLI in and of itself is often not a sufficient criterion for understanding whether it is “pathological” or “religious”; rather, a number of secondary and contextual factors are also involved in determining how to interpret DLI and how best to respond. Appraisals and responses to DLI are also inevitably shaped by the goals and expectations of meditation practitioners as well as by the worldviews and expertise of meditation teachers (Canby et al., 2025).

### The role of context in formation of delusion-like ideation

Many examples in our sample lend further support to the growing body of literature situating the genesis of delusional and other psychotic experiences in the social and experiential worlds in which they develop. Our participants were engaged in practices and were members of communities dedicated to spiritual and soteriological pursuits. They experienced significant disruptions to their normal lifestyles when in retreat, and they were often intrinsically motivated to embody and make sense of the norms, views, and values associated with Buddhist meditation. DLI that we report here illustrate some of the ways in which participants lived through challenging experiences. They may, on the one hand, reflect healthy coping strategies of making sense of existential questions and tasks that they are engaged with in meditation practice. This sense-making process can be temporarily adaptive or maladaptive. On the other hand, they may indicate potential vulnerability towards psychopathology. It is not known why some individuals react to stressors and challenges with DLIs, but our paper shows that these may be understood as spiritual idioms of distress.

Understanding DLI as a form of stress response for coping with challenges and sense-making has implications for a general framework of conceptualizing delusions and delusion-like ideation, both in the context of psychopathology and in non-clinical populations. The social (e.g., being in retreat), relational (e.g., hierarchical relationships with teachers), and practice-related (e.g., single-pointed concentration) aspects of meditation-related DLI reported here point to the embeddedness of DLI in complex embodied, existential, and social contexts. DLI reported by our participants, while generally falling within the common themes of delusions found cross-culturally, also often reflected specific aspects of Buddhist meditation culture. This demonstrates how individual distress is often expressed using idioms and metaphors located within the meaningful and local social world (Lewis-Fernández and Kirmayer, 2019) and is structured around local cultural affordances (Ramstead et al., 2016) that shape individual attention and express existential concerns using meaningful themes. Our examples illustrate the co-constitutive dynamics of social pressures, shared practices, environmental conditions, lived ontologies, and individual existential preoccupations in the genesis of meditation-related DLI.

### Limitations

The use of semi-structured interviews limits conclusions about frequency. Frequency of DLI among participants or the relative frequency of the types of DLI were based on spontaneous reporting rather than systematic querying and therefore do not necessarily reflect actual frequency. As a result, the DLI versus non-DLI comparisons should be understood as differences in groups that *reported* or *did not report* DLI. It is possible that the frequency of DLI or certain types of DLI would have been higher had systematic querying of each type been part of the interview or follow-up questionnaire, as open-ended queries are known to underestimate the frequency of adverse effects (Britton et al., 2021).

The sampling method for this exploratory study resulted in a heterogenous sample comprised of meditators of different ages and genders participating in a range of Buddhist traditions and lineages, at different intensities, and with different degrees of expertise. Other studies designed to control for specific demographic or practice-related variables might be able to find significant differences where we did not. Our sampling of Western Buddhists mainly in the United States also resulted in a sample dominantly comprised of white, convert practitioners. Accordingly, these findings may not be representative of the frequency of meditation-related DLI, its impacts, appraisals, and responses among other communities, such as Asian American Buddhists, or Buddhists in Asia, whether past or present.

The sampling and screening procedures also limit the conclusions that can be made about pre-existing mental health conditions. Meditation practitioners were excluded from analyses when a pre-existing condition could have accounted for the entire phenomenology of their challenges (whether DLI or other types of challenge) without meditation playing any causal or exacerbating role. Only three participants in the final dataset had serious pre-existing mental illness, and the two in the group reporting DLI made up only 6%, which is likely an underestimate of prevalence of psychopathology compared to an unrestricted, real-world sample. Similarly, meditators who have died or committed suicide following meditation-related DLI were not represented in our sample, but have been reported elsewhere (Kortava, 2021).

## Conclusion

In line with previous research, *The Varieties of Contemplative Experience* study finds that delusion-like ideation can arise in the context of meditation practice. This study identified eight distinct types of DLI that bear a resemblance to known types of DLI from research in the general population and across cultures. Impacts ranged from transient and insignificant to severe and debilitating, requiring removal from retreat, hospitalization and psychopharmacology. While some types of DLI are perhaps more immediately legible to meditation practitioners, teachers, or psychiatrists as problematic and warranting intervention, others—especially those presenting as special knowledge or related to death and dying—overlap with normative aspects of Buddhist meditation culture in such a way that they are subject to multiple possible interpretations and responses, often dependent on secondary factors such as associated distress, behavioral changes, or functional impairment.

Our work also provides further evidence for nascent theories of delusions and delusion-like experiences by suggesting that DLI is common in non-clinical populations, exists on a continuum of severity, and is not always associated with psychopathology. The content of DLI also tends to reflect and is sensitive to the social and cultural milieux in which it arises. For individuals navigating multiple cultures and epistemic frameworks, such as Western biomedical understanding of psychopathology as well as Buddhist philosophical and practical norms, DLI can be understood as spiritual idioms of distress, patterned by overlapping and sometimes conflicting worldviews, metaphors, and imagery.

Although there are many ways of contextualizing and explaining meditation-related DLI, as with other meditation-related challenges (Lindahl et al., 2019), a person-centered approach that takes into account the intersection of lived experience and cultural context is likely essential for understanding how best to interpret and respond to the challenges reported by meditation practitioners.

## Data Availability

The datasets presented in this article are not readily available because the data set is qualitative and contains sensitive information that cannot be fully de-identified. Requests to access the datasets should be directed to willoughby_britton@brown.edu.

## References

[ref1] Alle M. C. Berntsen D. (2021). Self-isolation, psychotic symptoms and cognitive problems during the COVID-19 worldwide outbreak. Psychiatry Res. 302:114015. doi: 10.1016/j.psychres.2021.114015, PMID: 34062477 PMC8131183

[ref2] American Psychiatric Association (2022). Diagnostic and statistical manual of mental disorders, fifth edition, text revision (DSM-5-TR). Washington: American Psychiatric Association.

[ref3] Arrigo B. A. Bullock J. L. (2008). The psychological effects of solitary confinement on prisoners in Supermax units: reviewing what we know and recommending what should change. Int. J. Offender Ther. Comp. Criminol. 52, 622–640. doi: 10.1177/0306624X07309720, PMID: 18025074

[ref4] Bar-el Y. Durst R. Katz G. Zislin J. Strauss Z. Knobler H. Y. (2000). Jerusalem syndrome. Br. J. Psychiatry 176, 86–90. doi: 10.1192/bjp.176.1.86, PMID: 10789334

[ref5] Bell V. Raihani N. Wilkinson S. (2021). Derationalizing delusions. Clin. Psychol. Sci. 9, 24–37. doi: 10.1177/2167702620951553, PMID: 33552704 PMC7820571

[ref6] Betts K. S. Scott J. Williams G. M. Najman J. M. Alati R. (2018). Validation of the importance of continua in representing delusional ideation in the general population. Schizophr. Res. 199, 304–312. doi: 10.1016/j.schres.2018.02.041, PMID: 29499964

[ref7] Bortolotti L. (2023). Why delusions matter. New York: Bloomsbury Academic.

[ref8] Bott N. Keller C. Kuppuswamy M. Spelber D. Zeier J. (2016). Cotard delusion in the context of schizophrenia: a case report and review of the literature. Front. Psychol. 7:1351. doi: 10.3389/fpsyg.2016.01351, PMID: 27656159 PMC5013050

[ref9] Britton W. B. Lindahl J. R. Cooper D. J. Canby N. K. Palitsky R. (2021). Defining and measuring meditation-related adverse effects in mindfulness-based programs. Clin. Psychol. Sci. 9, 1185–1204. doi: 10.1177/2167702621996340, PMID: 35174010 PMC8845498

[ref10] Cabrera H. L. Díaz-Garrido J. A. Martínez-Huidobro M. F. Medina T. R. (2024). Nuevas Adaptaciones Para la Aplicación del mindfulness a la Psicosis [new adaptations in the application of mindfulness to psychosis spectrum disorders]. Pap. Psicol. 45, 19–25.

[ref11] Canby N. K. Lindahl J. R. Cooper D. Joseph N. Palitsky R. Britton W. B. (2025). The teacher matters: the role of meditation teachers in the trajectories of Western Buddhist meditators experiencing meditation-related challenges. Contemp. Buddhism. 25, 9–53. doi: 10.1080/14639947.2025.2485677

[ref12] Chan-Ob T. Boonyanaruthee V. (1999). Meditation in association with psychosis. J. Med. Assoc. Thail. 82, 925–930, PMID: 10561951

[ref13] Charan D. Sharma P. Kachhawaha G. Kaur G. Gupta S. (2023). Meditation practices and the onset of psychosis: a case series and analysis of possible risk factors. Indian J. Psychol. Med. 45, 80–84. doi: 10.1177/02537176211059457, PMID: 36778606 PMC9896108

[ref14] Charlson F. J. Ferrari A. J. Santomauro D. F. Diminic S. Stockings E. Scott J. G.. (2018). Global epidemiology and burden of schizophrenia: findings from the global burden of disease study 2016. Schizophr. Bull. 44, 1195–1203. doi: 10.1093/schbul/sby058, PMID: 29762765 PMC6192504

[ref15] Ciaunica A. (2024). Selfless minds, unlimited bodies? Homeostatic bodily self-regulation in meditative experiences. J. Conscious. Stud. 31, 104–126. doi: 10.53765/20512201.31.5.104

[ref16] Cipriani G. Nuti A. Danti S. Picchi L. Di Fiorino M. (2019). ‘I am dead’: Cotard syndrome and dementia. Int. J. Psychiatry Clin. Pract. 23, 149–156. doi: 10.1080/13651501.2018.1529248, PMID: 30848970

[ref17] Collin S. Rowse G. Martinez A. P. Bentall R. P. (2023). Delusions and the dilemmas of life: a systematic review and meta-analyses of the global literature on the prevalence of delusional themes in clinical groups. Clin. Psychol. Rev. 104:102303. doi: 10.1016/j.cpr.2023.102303, PMID: 37390804

[ref18] Cook C. C. H. (2015). Religious psychopathology: the prevalence of religious content of delusions and hallucinations in mental disorder. Int. J. Soc. Psychiatry 61, 404–425. doi: 10.1177/0020764015573089, PMID: 25770205 PMC4440877

[ref19] Cooper D. J. Lindahl J. R. Palitsky R. Britton W. B. (2021). “Like a vibration cascading through the body”: energy-like somatic experiences reported by Western Buddhist meditators. Religion 12:1042. doi: 10.3390/rel12121042

[ref20] De Wit H. F. Baird M. L. (1991). Contemplative psychology. Pittsburgh: Duquesne University Press.

[ref21] Dihingia S. Bhuyan D. Bora M. Das N. (2023). Cotard’s delusion and its relation with different psychiatric diagnoses in a tertiary care hospital. Cureus 15:e39477. doi: 10.7759/cureus.39477, PMID: 37362522 PMC10290442

[ref22] Ellett L. (2024). Mindfulness for psychosis: current evidence, unanswered questions and future directions. Psychol. Psychother. 97, 34–40. doi: 10.1111/papt.12480, PMID: 37387330

[ref23] Ellis H. D. Young A. W. (1996). “Problems of person perception in schiziophrenia” in Schizophrenia - a neurophysiological perspectice. eds. Pantelis C. Nelson H. E. Barnes T. R. E. (Hoboken, NJ: John Wiley & Sons), 397–416.

[ref24] Farias M. Maraldi E. Wallenkampf K. C. Lucchetti G. (2020). Adverse events in meditation practices and meditation-based therapies: a systematic review. Acta Psychiatr. Scand. 142, 374–393. doi: 10.1111/acps.13225, PMID: 32820538

[ref25] Faunce A. F. Tennant W. B. (2024). Exploring Cotard’s delusion within the context of major depressive disorder with psychotic features: a case report. Cureus 16:e68047. doi: 10.7759/cureus.68047, PMID: 39347226 PMC11435227

[ref26] Freeman D. (2006). Delusions in the nonclinical population. Curr. Psychiatry Rep. 8, 191–204. doi: 10.1007/s11920-006-0023-1, PMID: 19817069

[ref27] Fusar-Poli P. Salazar de Pablo G. Rajkumar R. P. Lopez-Diaz A. Malhotra S. Heckers S.. (2022). Diagnosis, prognosis, and treatment of brief psychotic episodes: a review and research agenda. Lancet Psychiatry 9, 72–83. doi: 10.1016/S2215-0366(21)00121-8, PMID: 34856200

[ref28] Galante J. Montero-Marin J. Vainre M. Dufour G. Garcia-Campayo J. Jones P. B. (2024). Altered states of consciousness caused by a mindfulness-based programme up to a year later: results from a randomised controlled trial. PLoS One 19:e0305928. doi: 10.1371/journal.pone.0305928, PMID: 39018321 PMC11253948

[ref29] Gearing R. E. Alonzo D. Smolak A. McHugh K. Harmon S. Baldwin S. (2011). Association of religion with delusions and hallucinations in the context of schizophrenia: implications for engagement and adherence. Schizophr. Res. 126, 150–163. doi: 10.1016/j.schres.2010.11.005, PMID: 21131180

[ref30] Ghosh S. Chowdhury A. N. (2020). A case of two culture-bound syndromes (Koro and Dhat syndrome) coexisting with obsessive-compulsive disorder. Indian J. Psychiatry 62, 221–222. doi: 10.4103/psychiatry.IndianJPsychiatry_298_19, PMID: 32382189 PMC7197837

[ref31] Gold J. Gold I. (2015). Suspicious minds: Hoe culture shapes madness. New York: Simon and Schuster.

[ref32] Gold I. Gold J. (2025). “Culture and delusion” in The Routledge handbook of philosophy of delusion. ed. Sullivan-Besset E. (Abingdon: Routledge), 533–543.

[ref33] Goldberg S. B. Lam S. U. Britton W. B. Davidson R. J. (2021). Prevalence of meditation-related adverse effects in a population-based sample in the United States. Psychother. Res. 32, 291–305. doi: 10.1080/10503307.2021.1933646 34074221 PMC8636531

[ref34] Grunfeld G. Lemonde A. C. Gold I. Iyer S. N. Malla A. Lepage M.. (2023). “The more things change…”? Stability of delusional themes across 12 years of presentations to an early intervention service for psychosis. Soc. Psychiatry Psychiatr. Epidemiol. 58, 35–41. doi: 10.1007/s00127-022-02324-9, PMID: 35907013

[ref35] Hickey W. S. (2019). Mind cure: How meditation became medicine. Oxford: Oxford University Press.

[ref9003] Hodann-Caudevilla R. M. Díaz-Silveira C. Burgos-Julián F. A. Santed M. A. (2020). Mindfulness-Based Interventions for People with Schizophrenia: A Systematic Review and Meta-Analysis. International Journal of Environmental Research and Public Health, 17:4690. doi: 10.3390/ijerph17134690 32629764 PMC7369977

[ref36] Howes O. D. Whitehurst T. Shatalina E. Townsend L. Onwordi E. C. Mak T. L. A.. (2021). The clinical significance of duration of untreated psychosis: an umbrella review and random-effects meta-analysis. World Psychiatry 20, 75–95. doi: 10.1002/wps.20822, PMID: 33432766 PMC7801839

[ref37] Hufford D. J. (1989). The terror that comes in the night: An experience-centered study of supernatural assault traditions, vol. 7: University of Pennsylvania Press.

[ref38] Joshi S. Manandhar A. Sharma P. (2021). Meditation-induced psychosis: trigger and recurrence. Case Rep. Psychiatry 2021, 1–4. doi: 10.1155/2021/6615451, PMID: 34426774 PMC8380174

[ref39] Kaselionyte J. Gumley A. (2019). Psychosis or spiritual emergency? A foucauldian discourse analysis of case reports of extreme mental states in the context of meditation. Transcult. Psychiatry 56, 1094–1115. doi: 10.1177/1363461519861842, PMID: 31311435

[ref40] Kelleher I. Harley M. Murtagh A. Cannon M. (2011). Are screening instruments valid for psychotic-like experiences? A validation study of screening questions for psychotic-like experiences using in-depth clinical interview. Schizophr. Bull. 37, 362–369. doi: 10.1093/schbul/sbp057, PMID: 19542527 PMC3044617

[ref41] Kitayama S. Mesquita B. Karasawa M. (2006). Cultural affordances and emotional experience: socially engaging and disengaging emotions in Japan and the United States. J. Pers. Soc. Psychol. 91, 890–903. doi: 10.1037/0022-3514.91.5.890, PMID: 17059308

[ref9001] Kortava D. (2021). Lost in thought: the psychological risks of meditation. Harper’s Magazine, April 2021, 37–43. Available at: https://harpers.org/archive/2021/04/lost-in-thought-psychological-risks-of-meditation/

[ref42] Kuijpers H. J. van der Heijden F. M. Tuinier S. Verhoeven W. M. (2007). Meditation-induced psychosis. Psychopathology 40, 461–464. doi: 10.1159/000108125, PMID: 17848828

[ref43] Lambert D. van den Berg N. H. Mendrek A. (2021). Adverse effects of meditation: a review of observational, experimental and case studies. Curr. Psychol. 42, 1112–1125. doi: 10.1007/s12144-021-01503-2

[ref44] Laroi F. Luhrmann T. M. Bell V. Christian W. A. Jr. Deshpande S. Fernyhough C.. (2014). Culture and hallucinations: overview and future directions. Schizophr. Bull. 40, S213–S220. doi: 10.1093/schbul/sbu012 24936082 PMC4141319

[ref45] Lewis-Fernández R. Kirmayer L. J. (2019). Cultural concepts of distress and psychiatric disorders: understanding symptom experience and expression in context. Transcult. Psychiatry 56, 786–803. doi: 10.1177/1363461519861795, PMID: 31347476

[ref46] Lindahl J. R. (2017). Bodily energies and emotional traumas: a qualitative study of practice-related challenges reported by Vajrayāna Buddhists. Religion 8, 1–22. doi: 10.3390/rel8080153

[ref47] Lindahl J. R. Britton W. B. (2019). ‘I have this feeling of not really being there’: Buddhist meditation and changes in sense of self. J. Conscious. Stud. 26, 157–183.

[ref48] Lindahl J. R. Britton W. B. Cooper D. (2022a). Fear and terror in Buddhist meditation: a cognitive model for meditation-related changes in arousal and affect. J. Cogn. Historiogr. 7, 147–170. doi: 10.1558/jch.22807

[ref49] Lindahl J. R. Britton W. B. Cooper D. J. Kirmayer L. J. (2019). “Challenging and adverse meditation experiences: toward a person-centered approach” in The Oxford handbook of meditation. eds. Farias M. Brazier D. Lalljee M. (Oxford: Oxford University Press).

[ref50] Lindahl J. R. Cooper D. J. Fisher N. E. Kirmayer L. J. Britton W. B. (2020). Progress or pathology? Differential diagnosis and intervention criteria for meditation-related challenges: perspectives from Buddhist meditation teachers and practitioners. Front. Psychol. 11:1905. doi: 10.3389/fpsyg.2020.01905, PMID: 32849115 PMC7403193

[ref51] Lindahl J. R. Fisher N. E. Cooper D. J. Rosen R. K. Britton W. B. (2017). The varieties of contemplative experience: a mixed-methods study of meditation-related challenges in Western Buddhists. PLoS One 12:e0176239. doi: 10.1371/journal.pone.0176239, PMID: 28542181 PMC5443484

[ref52] Lindahl J. R. Kaplan C. T. Winget E. M. Britton W. B. (2014). A phenomenology of meditation-induced light experiences: traditional buddhist and neurobiological perspectives. Front. Psychol. 4:973. doi: 10.3389/fpsyg.2013.00973, PMID: 24427148 PMC3879457

[ref53] Lindahl J. R. Palitsky R. Cooper D. Britton W. B. (2022b). The roles and impacts of worldviews on the onset and trajectory of meditation-related challenges. Transcult. Psychiatry 60, 637–650. doi: 10.1177/1363461522112867 36476189 PMC11292974

[ref54] Lingpa D. (2011). A clear mirror: The visionary autobiography of a Tibetan master. Kathmandu: Rangjung Yeshe Publications.

[ref55] Lingpa D. (2015). The Vajra essence: Düdjom Lingpa’s visions of the great perfection (Vol. 3). Somerville, MA: Wisdom Publications.

[ref56] Luhrmann T. M. Padmavati R. Tharoor H. Osei A. (2015). Hearing voices in different cultures: a social kindling hypothesis. Top. Cogn. Sci. 7, 646–663. doi: 10.1111/tops.12158, PMID: 26349837

[ref57] Maher B. A. (1974). Delusional thinking and perceptual disorder. J. Individ. Psychol. 30, 98–113, PMID: 4857199

[ref58] Maher B. A. (1999). Anomalous experience in everyday life: its significance for psychopathology. Monist 82, 547–570. doi: 10.5840/monist199982428

[ref59] Mason O. J. Brady F. (2009). The psychotomimetic effects of short-term sensory deprivation. J. Nerv. Ment. Dis. 197, 783–785. doi: 10.1097/NMD.0b013e3181b9760b, PMID: 19829208

[ref60] McCreery C. Claridge G. (2002). Healthy schizotypy: the case of out-of-the-body experiences. Pers. Individ. Differ. 32, 141–154. doi: 10.1016/S0191-8869(01)00013-7

[ref61] McMahan D. L. (2008). The making of Buddhist modernism. Oxford: Oxford University Press.

[ref62] McMahan D. L. Braun E. (2017). Meditation, Buddhism, and science. Oxford: Oxford University Press.

[ref63] Mianji F. Semnani Y. (2015). Zar spirit possession in Iran and African countries: group distress, culture-bound syndrome or cultural concept of distress? Iran. J. Psychiatry 10, 225–232, PMID: 27006667 PMC4801492

[ref64] Morgan C. MacKenzie K. Fearon P. (2008). Society and psychosis. Cambridge: Cambridge University Press.

[ref65] Murphy D. (2015). ““Deviant deviance”: cultural diversity in DSM-5” in The DSM-5 in perspective. eds. Demazeux S. Singy P. (Princeton: Springer), 97–110.

[ref66] Nichter M. (2010). Idioms of distress revisited. Cult. Med. Psychiatry 34, 401–416. doi: 10.1007/s11013-010-9179-6, PMID: 20495999

[ref67] O’Brien-Venus B. Ellett L. Burgess-Barr S. (2024). Systematic review of the safety of mindfulness-based interventions for psychosis. Clin. Psychol. Rev. 112:102445. doi: 10.1016/j.cpr.2024.102445, PMID: 38851179

[ref68] Palacios-Sanchez L. Botero-Meneses J. S. Pachon R. P. Hernandez L. B. P. Triana-Melo J. D. P. Ramirez-Rodriguez S. (2018). Stendhal syndrome: a clinical and historical overview. Arq. Neuropsiquiatr. 76, 120–123. doi: 10.1590/0004-282x20170189, PMID: 29489968

[ref69] Pappa E. Baah F. Lynch J. Shiel L. Blackman G. Raihani N.. (2025). Delusional themes are more varied than previously assumed: a comprehensive systematic review and Meta-analysis. Schizophr. Bull. 51, 637–645. doi: 10.1093/schbul/sbae225, PMID: 39847500 PMC12061659

[ref70] Pauly L. Bergmann N. Hahne I. Pux S. Tam Ta T. M. Rapp M.. (2022). Prevalence, predictors and types of unpleasant and adverse effects of meditation in regular meditators: international cross-sectional study. BJPsych Open 8, 1–8. doi: 10.1192/bjo.2021.106635361299

[ref71] Picardi A. Fonzi L. Pallagrosi M. Gigantesco A. Biondi M. (2018). Delusional themes across affective and non-affective psychoses. Front. Psychol. 9:132. doi: 10.3389/fpsyt.2018.00132, PMID: 29674982 PMC5895977

[ref72] Pillmann F. Marneros A. (2003). Brief and acute psychoses: the development of concepts. Hist. Psychiatry 14, 161–177. doi: 10.1177/0957154X030142002, PMID: 14518487

[ref73] Prakash R. Aggarwal N. Kataria D. Prasad S. (2018). Meditation induced psychosis: case report. Asian J. Psychiatr. 31, 109–110. doi: 10.1016/j.ajp.2018.02.001, PMID: 29475163

[ref74] Radua J. Ramella-Cravaro V. Ioannidis J. P. A. Reichenberg A. Phiphopthatsanee N. Amir T.. (2018). What causes psychosis? An umbrella review of risk and protective factors. World Psychiatry 17, 49–66. doi: 10.1002/wps.20490, PMID: 29352556 PMC5775150

[ref75] Ramstead M. J. Veissiere S. P. Kirmayer L. J. (2016). Cultural affordances: scaffolding local worlds through shared intentionality and regimes of attention. Front. Psychol. 7:1090. doi: 10.3389/fpsyg.2016.01090, PMID: 27507953 PMC4960915

[ref76] Robertson K. Gold I. Veissiere S. Robillard R. Solomonova E. (2024). Delusional ideation is associated with social imagery: felt presence, social anxiety, empathy and loneliness. Psychiatr. Res. Commun. 4:100169. doi: 10.1016/j.psycom.2024.100169

[ref77] Sahin F. Candansayar S. Genis B. (2022). Revisiting Jerusalem syndrome: a case displaying similar symptoms to Jerusalem syndrome during Mecca visit. Turk Psikiyatri Derg. 33, 290–292. doi: 10.5080/u26966, PMID: 36592108

[ref78] Salguero C. P. (2023). “Meditation sickness” in medieval Chinese Buddhism and the contemporary west. J. Buddh. Ethics 30, 169–211.

[ref79] Sayadaw M. s. (1965). The progress of insight: A treatise on Satipattḥāna meditation. (Nyā naponika Thera)

[ref80] Sethi S. Bhargava S. C. (2003). Relationship of meditation and psychosis: case studies. Aust. N. Z. J. Psychiatry 37:382. doi: 10.1046/j.1440-1614.2003.11721.x, PMID: 12780479

[ref81] Shapiro D. H. Jr. Giber D. (1978). Meditation and psychotherapeutic effects. Self-regulation strategy and altered state of consciousness. Arch. Gen. Psychiatry 35, 294–302. doi: 10.1001/archpsyc.1978.01770270044003, PMID: 365121

[ref82] Sharma P. Mahapatra A. Gupta R. (2019). Meditation-induced psychosis: a narrative review and individual patient data analysis. Ir. J. Psychol. Med. 39, 391–397. doi: 10.1017/ipm.2019.47, PMID: 31668156

[ref83] Sharma P. Singh S. Gnanavel S. Kumar N. (2016). Meditation - a two edged sword for psychosis: a case report. Ir. J. Psychol. Med. 33, 247–249. doi: 10.1017/ipm.2015.73, PMID: 30115157

[ref84] Siddle R. Haddock G. Tarrier N. Faragher E. B. (2014). Religious delusions in patients admitted to hospital with schizophrenia. Soc. Psychiatry Psychiatr. Epidemiol. 37, 130–138. doi: 10.1007/s001270200005, PMID: 11990010

[ref85] Smeets F. Lataster T. van Winkel R. de Graaf R. Ten Have M. van Os J. (2013). Testing the hypothesis that psychotic illness begins when subthreshold hallucinations combine with delusional ideation. Acta Psychiatr. Scand. 127, 34–47. doi: 10.1111/j.1600-0447.2012.01888.x, PMID: 22676336

[ref86] Smith P. S. (2006). The effects of solitary confinement on prison inmates: a brief history and review of the literature. Crime Just. Rev. Res. 34, 441–528. doi: 10.1086/500626

[ref87] Sparby T. Eilinghoff-Ehlers P. Lewandowski N. Pachernegg Y. Schnitzler L. Edelhauser F. (2024). Meditation hindrances and breakthroughs: a multilevel first-person phenomenological analysis. Religion 15:865. doi: 10.3390/rel15070865

[ref88] Suhail K. Cochrane R. (2002). Effect of culture and environment on the phenomenology of delusions and hallucinations. Int. J. Soc. Psychiatry 48, 126–138. doi: 10.1177/002076402128783181, PMID: 12182508

[ref89] Taylor G. B. Vasquez T. S. Kastrinos A. Fisher C. L. Puig A. Bylund C. L. (2022). The adverse effects of meditation-interventions and mind–body practices: a systematic review. Mindfulness 13, 1839–1856. doi: 10.1007/s12671-022-01915-6

[ref90] The Śūraṅgama Sūtra (1966). London: Rider.

[ref91] Van Dam N. T. Targett J. Davies J. N. Burger A. Galante J. (2025). Incidence and predictors of meditation-related unusual experiences and adverse effects in a representative sample of meditators in the United States. Clin. Psychol. Sci. 13, 632–648. doi: 10.1177/21677026241298269

[ref92] Van Dam N. T. van Vugt M. K. Vago D. R. Schmalzl L. Saron C. D. Olendzki A.. (2018). Mind the hype: a critical evaluation and prescriptive agenda for research on mindfulness and meditation. Perspect. Psychol. Sci. 13, 36–61. doi: 10.1177/1745691617709589, PMID: 29016274 PMC5758421

[ref93] Wilson J. (2014). Mindful America: Meditation and the mutual transformation of Buddhism and American culture. USA: Oxford University Press.

[ref94] Yeager S. M. (2019). “The earthly and heavenly Jerusalem” in The Cambridge companions to the literature of the crusades. ed. Bale A. (Cambridge: Cambridge University Press).

[ref9004] Yip A. L. K. Karatzias T. Chien W. T. (2024). Mindfulness-based interventions for non-affective psychosis: a comprehensive systematic review and meta-analysis, Annals of Medicine, 54, 2339–2352, doi: 10.1080/07853890.2022.2108551 PMC9423825 36004784

[ref95] Yung A. R. Lin A. (2016). Psychotic experiences and their significance. World Psychiatry 15, 130–131. doi: 10.1002/wps.20328, PMID: 27265701 PMC4911755

